# Predicting immune responsiveness in ER-positive breast cancer for personalized therapy: a population-based study

**DOI:** 10.1038/s41698-025-01035-z

**Published:** 2025-07-23

**Authors:** Axel Stenmark Tullberg, Sara Woxlin, Filippa Sjölin, Ella Ittner, Anikò Kovàcs, Khalil Helou, Erik Holmberg, Per Karlsson

**Affiliations:** 1 Department of Oncology, Institute of Clinical Sciences, Sahlgrenska Academy, University of Gothenburg, Sahlgrenska University Hospital, Gothenburg, Sweden; 2 Division of Oncology, Stanford University, Stanford, CA USA; 3 Department of Clinical Pathology, Sahlgrenska University Hospital, Gothenburg, Sweden

**Keywords:** Cancer, Breast cancer, Tumour immunology, Predictive markers, Prognostic markers

## Abstract

The immune system’s role in estrogen receptor (ER)-positive breast cancer is poorly understood. A population-based cohort of 428 breast cancer patients with clinical and molecular data was analyzed to assess how immune biomarkers can inform treatment decisions. Tumor-intrinsic immune responsiveness and local immune infiltration were quantified, and epithelial cell states were derived using EcoTyper. The interaction between ProliferativeIndex and Immunescore predicted risk of local recurrence in ER-positive tumors (HR 0.56, 95% CI 0.36–0.88, *p* = 0.012). EcoTyper identified two epithelial cell states, S04 and S05, with distinct immunomodulatory properties. S04 tumors showed higher proliferation, enrichment for M1 macrophages, CD8 effector T-cells, and plasma cells, alongside hypomethylation of immune-related pathways and hypermethylation of the PI3K signaling pathway. In contrast, S05-enriched tumors were associated with fibroblast activation, immune exclusion, and enrichment for glycosylation-related pathways. These findings suggest that epithelial cell states shape immune responsiveness in ER-positive breast cancer and may inform biomarker-driven treatment strategies.

## Introduction

The immune infiltrate plays an important role in breast cancer, influencing both prognosis and treatment response^1^. The prognostic and predictive value is well-established in HER2-positive and triple-negative subtypes, where tumor-infiltrating lymphocytes (TILs) consistently associate with favorable outcomes^2^. In contrast, the role of immune infiltration in estrogen receptor-positive (ER-positive) breast cancer is less clear. In this setting, immune biomarkers have shown inconsistent associations with prognosis^3–7^, reflecting the complex interplay between tumor biology and the immune microenvironment. Simply quantifying TILs is insufficient for risk stratification, making it crucial to elucidate underlying biological determinants that shape immune responsiveness. Immune biomarkers hold promise for risk stratification in ER-positive disease^8^, but more evidence is needed to guide clinical decision-making. Proliferation appears to be a key determinant of immune competence^9^, supported by the association between high-risk features and immunotherapy benefit in ER-positive tumors^10,11^. A deeper understanding of how tumor-intrinsic traits regulate immune activation would improve risk stratification and may provide biomarkers or therapeutic targets for immune-targeted treatments.

Postoperative radiotherapy (RT) is an area with an unmet need for biomarkers that can guide treatment decisions. The ability to identify patients who can safely be omitted from RT could significantly transform patient care. Moreover, traditionally regarded high-risk patients with immune-infiltrated tumors might benefit from adjusted RT doses rather than the standard boost used in higher-risk settings^9^. Trials such as PRIME II have investigated the omission of RT in older women with clinically low-risk ER-positive tumors^12^. The POLAR and ARTIC studies have made strides to incorporate genetic profiles into RT decision-making^13–15^. However, these efforts primarily relied on clinical/genomic markers of tumor aggressiveness and did not specifically investigate the influence of the local immune infiltrate.

The current study expands on previous research by investigating how immune infiltration can be used to guide risk stratification in early breast cancer^9,16,17^. To quantify immune infiltration and tumor-intrinsic immune responsiveness, we previously developed Immunescore and ProliferativeIndex, two gene expression–based signatures. Immunescore was designed to capture immune infiltration, while ProliferativeIndex reflects tumor-intrinsic features associated with immune responsiveness. Together, they provide a more nuanced view of the tumor–immune interface. Using a population-based cohort, we sought to validate this framework specifically in ER-positive disease. In addition, we apply EcoTyper, a framework that infers cell states from transcriptional programs, to further investigate tumor-intrinsic phenotypes and their relationships with the tumor microenvironment (TME)^18^. We hypothesized that distinct tumor-intrinsic epithelial traits influence the immune microenvironment and may serve as biomarkers for personalized treatment decisions.

## Results

### Descriptive Results for the JK Biobank Population-Based Cohort

**Fig. 1:**
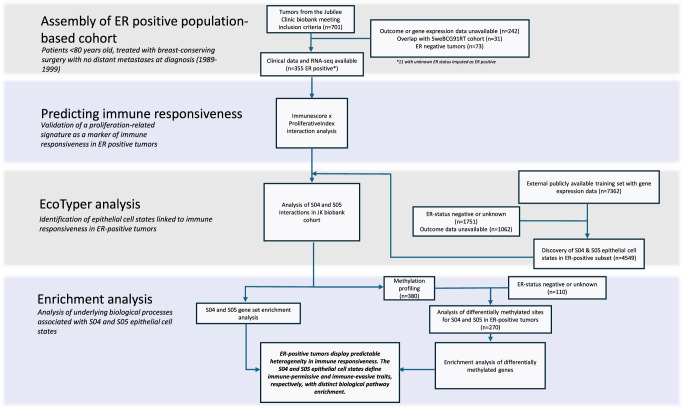
STARD diagram including steps from assembly of the Jubilee Clinic (JK) biobank cohort to analyses. A population-based cohort with long-term follow-up was assembled and molecular and clinical data were obtained. The aim of the study was to identify predictors of immune responsiveness in ER-positive breast cancer and to deepen the understanding of the underlying biological processes to provide a framework for prognostic and potentially treatment-predictive patient stratification.

Descriptive statistics are presented for the imputed data set used in all analyses (Table 1) while pre-imputation data are presented in Supplementary Data S1. After imputations, a total of 428 tumors with available clinical and molecular data were included in the study (418 fresh-frozen and 10 FFPE), Fig. 1. Of these, 299 patients received RT, 214 received endocrine treatment, and 147 received chemotherapy. Patients receiving RT were more likely to have undergone axillary lymph node dissection (93.0% vs. 57.4%, *p* < 0.001), were more likely to have lymph node metastases (median 1 vs. 0, *p* = 0.001), and had a lower median age (53 years vs. 70 years, *p* < 0.001). In total, 355 (82.9%) tumors were ER-positive and 273 (63.8%) were progesterone receptor (PgR)-positive. The median tumor size was 21 mm, a total of 30 tumors had positive margins, and the median age was 58 years, Table 1. After imputing missing values for histological grade (*n* = 88, 20.6%, Supplementary Data S2), ER (*n* = 10, 2.1%), and PgR (*n* = 11, 2.3%), 211 (49.3%) were classified as Luminal A, 126 (29.4%) as Luminal B, 35 (8.2%) as HER2-positive, and 56 (13.1%) as non-luminal. When comparing pathological subtype classification to PAM50-like classification, 99.1% of tumors classified as either Luminal A or B using the PAM50-like algorithm were classified as ER-positive, Supplementary Data S3. Immunescore and ProliferativeIndex were significantly associated with higher histological grade (rho 0.37, *p* < 0.001; rho 0.57, *p* < 0.001), lymph node positivity (rho 0.16, *p* = 0.002; rho 0.20, *p* < 0.001), and ESR1 expression (rho: -0.34 for both, *p* < 0.001). ProliferativeIndex was also associated with increased tumor size (rho 0.34, *p* < 0.001). Both scores showed weak negative associations with age (rho -0.16, *p* < 0.001; rho -0.12, *p* = 0.009). Unless otherwise specified, all subsequent analyses are restricted to tumors classified as ER-positive after imputation.

**Table 1:** Demographics of the JK biobank cohort after imputations

		Estrogen Receptor Positive				Estrogen Receptor Negative				All Tumors			
Variable	Category	No RT	RT	All	*P* value	No RT	RT	All	*P* value	No RT	RT	All	*P* value
Axillary status	No palpable nodes	105 (92.1%)	220 (91.3%)	325 (91.5%)		15 (100%)	48 (82.8%)	63 (86.3%)		120 (93%)	268 (89.6%)	388 (90.7%)	
	Mobile lymph nodes	8 (7.0%)	21 (8.7%)	29 (8.2%)		0 (0%)	10 (17.2%)	10 (13.7%)		8 (6.2%)	31 (10.4%)	39 (9.1%)	
	Fixed lymph nodes	1 (0.9%)	0 (0%)	1 (0.3%)	0.36	0 (0%)	0 (0%)	0 (0%)	0.11	1 (0.8%)	0 (0%)	1 (0.2%)	0.11
Axillary surgery	No	48 (42.1%)	19 (7.9%)	67 (18.9%)		7 (46.7%)	2 (3.4%)	9 (12.3%)		55 (42.6%)	21 (7.0%)	76 (17.8%)	
	Yes	66 (57.9%)	222 (92.1%)	288 (81.1%)	<0.01	8 (53.3%)	56 (96.6%)	64 (87.7%)	<0.01	74 (57.4%)	278 (93.0%)	352 (82.2%)	<0.01
ER status	Positive	114 (100%)	241 (100%)	355 (100%)		0 (0%)	0 (0%)	0 (0%)		114 (88.4%)	241 (80.6%)	355 (82.9%)	
	Negative	0 (0%)	0 (0%)	0 (0%)	1.00	15 (100%)	58 (100%)	73 (100%)	1.00	15 (11.6%)	58 (19.4%)	73 (17.1%)	0.05
PgR status	Positive	90 (78.9%)	178 (73.9%)	268 (75.5%)		2 (13.3%)	3 (5.2%)	5 (6.8%)		92 (71.3%)	181 (60.5%)	273 (63.8%)	
	Negative	24 (21.1%)	63 (26.1%)	87 (24.5%)	0.36	13 (86.7%)	55 (94.8%)	68 (93.2%)	0.27	37 (28.7%)	118 (39.5%)	155 (36.2%)	0.04
Endocrine therapy	No	45 (39.5%)	111 (46.1%)	156 (43.9%)		11 (73.3%)	47 (81.0%)	58 (79.5%)		56 (43.4%)	158 (52.8%)	214 (50.0%)	
	Yes	69 (60.5%)	130 (53.9%)	199 (56.1%)	0.25	4 (26.7%)	11 (19.0%)	15 (20.5%)	0.49	73 (56.6%)	141 (47.2%)	214 (50.0%)	0.09
Chemotherapy	No	103 (90.4%)	156 (64.7%)	259 (73.0%)		8 (53.3%)	14 (24.1%)	22 (30.1%)		111 (86.0%)	170 (56.9%)	281 (65.7%)	
	Yes	11 (9.6%)	85 (35.3%)	96 (27.0%)	<0.01	7 (46.7%)	44 (75.9%)	51 (69.9%)	0.05	18 (14.0%)	129 (43.1%)	147 (34.3%)	<0.01
Histological grade	1	23 (20.2%)	51 (21.2%)	74 (20.8%)		2 (13.3%)	5 (8.6%)	7 (9.6%)		25 (19.4%)	56 (18.7%)	81 (18.9%)	
	2	59 (51.8%)	119 (49.4%)	178 (50.1%)		2 (13.3%)	13 (22.4%)	15 (20.5%)		61 (47.3%)	132 (44.1%)	193 (45.1%)	
	3	32 (28.1%)	71 (29.5%)	103 (29%)	0.92	11 (73.3%)	40 (69.0%)	51 (69.9%)	0.65	43 (33.3%)	111 (37.1%)	154 (36.0%)	0.76
Radical surgery	No	11 (9.6%)	15 (6.2%)	26 (7.3%)		1 (6.7%)	3 (5.2%)	4 (5.5%)		12 (9.3%)	18 (6%)	30 (7.0%)	
	Yes	103 (90.4%)	226 (93.8%)	329 (92.7%)	0.28	14 (93.3%)	55 (94.8%)	69 (94.5%)	1.00	117 (90.7%)	281 (94%)	398 (93.0%)	0.22
Subtype	LumA	68 (59.6%)	143 (59.3%)	211 (59.4%)		0 (0%)	0 (0%)	0 (0%)		68 (52.7%)	143 (47.8%)	211 (49.3%)	
	LumB	42 (36.8%)	84 (34.9%)	126 (35.5%)		0 (0%)	0 (0%)	0 (0%)		42 (32.6%)	84 (28.1%)	126 (29.4%)	
	HER2-positive	4 (3.5%)	14 (5.8%)	18 (5.1%)	0.68	5 (33.3%)	12 (20.7%)	17 (23.3%)		9 (7.0%)	26 (8.7%)	35 (8.2%)	
	Non-luminal	0 (0%)	0 (0%)	0 (0%)		10 (66.7%)	46 (79.3%)	56 (76.7%)	0.32	10 (7.8%)	46 (15.4%)	56 (13.1%)	0.14
Age (years)	Median (SD)	71 (11.6)	54 (11.41)	59 (12.56)	<0.01	64 (12.51)	50 (11.02)	52 (11.97)	0.01	70 (11.74)	53 (11.39)	58 (12.58)	<0.01
Size (mm)	Median (SD)	21 (12.39)	20 (11.23)	21 (11.60)	0.64	23 (11.61)	25 (11.60)	25 (11.59)	0.38	21 (12.26)	21 (11.42)	21 (11.66)	0.89
Number of positive lymph nodes	Median (SD)	0 (3.31)	1 (3.17)	1 (3.22)	<0.01	1 (1.98)	1 (4.42)	1 (4.05)	0.71	0 (3.18)	1 (3.44)	1 (3.37)	<0.01
Follow-up time*	Median (SD)	5.95 (5.72)	10.23 (6.62)	9.07 (6.52)	<0.01	3.85 (7.65)	5.48 (7.76)	5.06 (7.70)	0.47	5.83 (5.94)	9.88 (6.9)	8.76 (6.74)	<0.01
IBTR event	No Event	11 (9.6%)	72 (29.9%)	83 (23.4%)		2 (13.3%)	22 (37.9%)	24 (32.9%)		13 (10.1%)	94 (31.4%)	107 (25%)	
	Event	31 (27.2%)	43 (17.8%)	74 (20.8%)		6 (40.0%)	5 (8.6%)	11 (15.1%)		37 (28.7%)	48 (16.1%)	85 (19.9%)	
	Competing Event	72 (63.2%)	126 (52.3%)	198 (55.8%)	<0.01	7 (46.7%)	31 (53.4%)	38 (52.1%)	0.64	79 (61.2%)	157 (52.5%)	236 (55.1%)	<0.01

Demographics of the population-based JK biobank cohort after imputations. This is the cohort used for analysis. For pre-imputation demographics including missing values, please refer to Supplementary Data S1 and S2. The non-luminal subtype was defined as ER-negative and HER2-negative. A total of 5 of these were PgR positive. ER= Estrogen Receptor. PgR= Progesterone Receptor. *Cox regression and flexible parametrics analyses were restricted to 10 years of follow up resulting in similar follow-up time between the RT and no RT (5.92 vs 6.96 years, *p* = 0.64) arms.

### Prognostic Effect of Immunescore

In univariable analysis, higher histological grade (histological grade 3 versus 1, HR 2.4 CI 95% 1–5.76, *p* = 0.049 and histological grade 2 versus 1, HR 1.52 CI 95% 0.65-3.53, *p* = 0.33), subtype (luminal B vs luminal A; HR 2.12, CI 95% 1.18–3.84, *p* = 0.013; HER2-positive vs luminal A, HR 4.51 CI 95% 1.7–11.99, *p* = 0.003), and increased tumor size (HR 1.03, 95% CI: 1.01–1.05 per mm, *p* = 0.003) were associated with a higher risk of ipsilateral breast tumor recurrence (IBTR). RT (HR 0.37, 95% CI: 0.21–0.65, *p* < 0.001) and radical surgery (HR 0.22 CI 95% 0.10–0.50, *p* < 0.001) were associated with a decreased IBTR risk, Table 2. The interaction between Immunescore and ProliferativeIndex was borderline significant in univariable analysis of the ER-positive subset (HR 0.68, CI 95% 0.46–1.0, *p* = 0.050) and significant in analysis of all tumors (HR 0.63, 95% CI: 0.47–0.84, *p* = 0.001), Supplementary Data S4.

**Table 2:** Uni- and multivariable analysis of the risk of ipsilateral breast tumor recurrence in estrogen receptor positive tumors in the JK biobank cohort

Covariates		Univariable		Multivariable	
		HR (CI 95%)	*P* value	HR (CI 95%)	*P* value
	Immunescore	1.04 (95% CI: 0.75–1.43)	0.817	1.07 (95% CI: 0.75–1.52)	0.719
	ProliferativeIndex	1.74 (95% CI: 1.24–2.44)	0.001	1.6 (95% CI: 1.03–2.49)	0.035
ProliferativeIndex x Immunescore interaction	Immunescore x ProliferativeIndex	0.68 (95% CI: 0.46–1)	0.05	0.56 (95% CI: 0.36–0.88)	0.012
	No	1.0 (reference)		1.0 (reference)	
Radiotherapy	Yes	0.37 (95% CI: 0.21–0.65)	<0.001	0.31 (95% CI: 0.17–0.57)	<0.001
	No	1.0 (reference)		1.0 (reference)	
Radical surgery	Yes	0.22 (95% CI: 0.1–0.5)	<0.001	0.31 (95% CI: 0.13–0.72)	0.006
Tumor size (mm)		1.03 (95% CI: 1.01–1.05)	0.003	1.02 (95% CI: 1–1.05)	0.036
	No	1.0 (reference)		1.0 (reference)	
Systemic treatment	Yes	0.66 (95% CI: 0.38–1.17)	0.153	0.37 (95% CI: 0.2–0.69)	0.002
	Luminal A	1.0 (reference)		1.0 (reference)	
Subtype	Luminal B	2.12 (95% CI: 1.18–3.84)	0.013	1.56 (95% CI: 0.77-3.14)	0.215
	HER2-positive	4.51 (95% CI: 1.7–11.99)	0.003	4.45 (95% CI: 1.51–13.1)	0.007
	No	1.0 (reference)			
Endocrine therapy	Yes	0.75 (95% CI: 0.43–1.32)	0.321		
	No	1.0 (reference)			
Chemotherapy	Yes	0.87 (95% CI: 0.46–1.67)	0.683		
	1	1.0 (reference)			
	2	1.52 (95% CI: 0.65–3.53)	0.33		
Histological grade	3	2.4 (95% CI: 1–5.76)	0.049		
Number of positive lymph nodes		1.04 (95% CI: 0.95–1.14)	0.395		
Age (years)		0.99 (95% CI: 0.97–1.01)	0.483		
	Positive	1.0 (reference)			
PgR	Negative	1.22 (95% CI: 0.65–2.3)	0.538		

Uni- and multivariable analysis of ER-positive tumors. Interaction tests were performed using the likelihood ratio test. The endpoint used was ipsilateral breast tumor recurrence (IBTR). PgR= Progesterone Receptor.

**Fig. 2:**
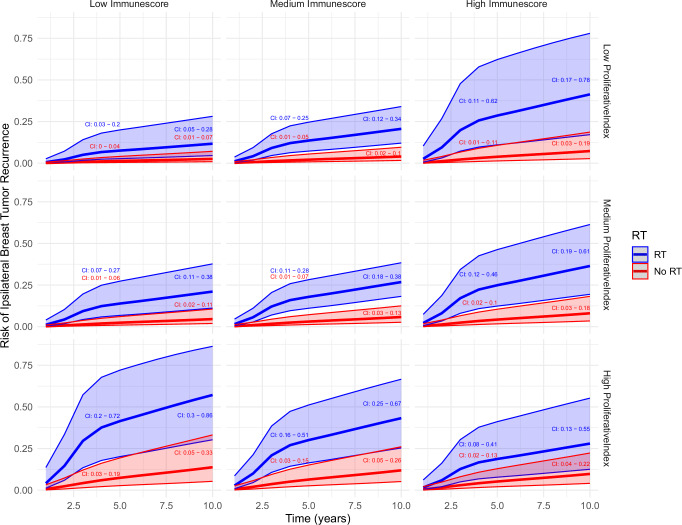
Flexible parametrics modeling of predicted IBTR risk in ER-positive tumors depending on Immunescore and ProliferativeIndex. Predicted cumulative incidence of ipsilateral breast tumor recurrence (IBTR) depending on Immunescore and ProliferativeIndex in ER-positive tumors with and without radiotherapy. High, low, and medium values were generated using the median of tumors within the lowest quartile, overall median, and median of tumors within the highest quartile for Immunescore and ProliferativeIndex. Separate models were fitted for irradiated and unirradiated patients. Numbers represent 95% confidence intervals at 5- and 10-year marks. The immune infiltrate, as measured by Immunescore, had a favorable prognostic effect primarily in patients with tumors with high ProliferativeIndex. Full models are available in the supplement.

The following multivariable analysis was adjusted for subtype, RT, radical surgery, tumor size, and systemic therapy. Radical surgery (HR 0.31 CI 95% 0.13–0.72, *p* = 0.006), RT (HR 0.31 CI 95% 0.17-0.57, *p* < 0.001), and systemic therapy (HR 0.37 CI 95% 0.20–0.69, *p* = 0.002) were associated with a decreased risk of IBTR. In contrast, tumor size (HR 1.02, CI 95% 1.0–1.05, *p* = 0.036) and subtype (HER2-positive vs luminal A, HR 4.45, CI 95% 1.51–13.1, *p* = 0.007) were associated with increased IBTR risk. The interaction between Immunescore and ProliferativeIndex was significant in both the ER-positive subset (HR 0.56, 95% CI: 0.36–0.88, *p* = 0.012) and all tumors (HR 0.61, CI 95% 0.43–0.87, *p* = 0.004), suggesting that the interplay between proliferation and immune infiltration influences prognosis in ER-positive disease. Flexible parametric modeling of ER-positive tumors predicted that low Immunescore and low ProliferativeIndex had the most favorable prognosis while increasing Immunescore was beneficial only in tumors with high ProliferativeIndex, Fig. 2, Supplementary Data S5 and Supplementary Data S6.

### Stratification of high-risk tumors by Immunescore

Given that RT boost is routinely recommended for high-risk patients^19,20^, we investigated whether Immunescore could identify patients, regardless of ER status, with a favorable prognosis despite not receiving a boost. Patients <50 years of age treated with RT, systemic treatment, and radical surgery and patients 50–65 years with histological grade 3 tumors treated with RT, systemic treatment, and radical surgery were selected based on current guidelines^19,20^ and split by the median Immunescore within each group. Patients with an Immunescore below the 50th percentile had a cumulative IBTR incidence of 22.2% (CI: 10.0–34.3%) at 15 years, while those above the median had a cumulative incidence of 11.6% (CI: 1.7–21.5.0%), despite no RT boost being administered, *p* = 0.18, Figure S1.

### Epithelial Cell States Associated with Benefit from an Immune Response

To better understand how tumor-intrinsic characteristics influence immune responses in ER-positive tumors, we analyzed 4549 ER-positive tumors from 21 publicly available data sets. Using EcoTyper, we profiled six previously defined epithelial cell states (S01-S06) based on large scale pan-cancer analysis^18^. These states capture distinct transcriptional programs within the epithelial compartment, including basal-like (S01), normal-enriched (S02), pro-angiogenic (S03), pro-inflammatory (S04), unknown (S05), and metabolic (S06) phenotypes. The S05 and S06 cell states appeared normally distributed and were most abundant, whereas S01-S04 were positively skewed, Figure S2. Cox regressions revealed that the S04 state enhanced the prognostic effect of Immunescore (Padj_interaction_= 0.009), while S05 blunted this effect (Padj_interaction_ = 0.046), Table 3. The other cell states were non-informative in interaction analysis, and thus downstream analyses focused on the S04 and S05 epithelial cell states.

**Table 3:** Analysis of immunomodulatory effects of epithelial cell states in test and validation cohorts

	Training cohort		ER-positive subset validation				All patients validation			
Term	HR (CI 95%) training	P adj	HR (CI 95%) uni ER pos	P univ ER pos	HR (CI 95%) multiv ER pos	P multiv ER pos	HR (CI 95%) univ all	P univ all	HR (CI 95%) multiv all	P multiv all
Epithelial S01 state	0.96 (0.9-1.03)	0.296								
Epithelial S02 state	1.04 (0.97-1.12)	0.296								
Epithelial S03 state	1.01 (0.94-1.08)	0.896								
Epithelial S04 state	0.93 (0.88-0.98)	0.009	0.67 (0.46-1)	0.048	0.74 (0.5-1.1)	0.134	0.62 (0.43-0.89)	0.009	0.69 (0.49-0.99)	0.043
Epithelial S05 state	1.07 (1.01-1.14)	0.046	1.47 (1.01-2.14)	0.046	1.62 (1.11-2.35)	0.011	1.64 (1.23-2.2)	0.001	1.82 (1.34-2.48)	<0.001
Epithelial S06 state	1.04 (0.98-1.1)	0.288								

Interaction tests between epithelial cell states and Immunescore in training cohort, ER-positive subset of JK biobank validation cohort, and the whole JK biobank validation cohort. Analyses in the training cohort included ProliferativeIndex to adjust for proliferation. Multivariable analyses in the validation cohort were adjusted for subtype, systemic therapy, tumor size, radiotherapy, radicality of surgery, and ProliferativeIndex. The epithelial phenotypes S04 and S05 had opposing interaction effects on Immunescore, indicating opposite immunomodulatory effects. The endpoint used for the validation analysis in the JK biobank cohort was ipsilateral breast tumor recurrence (IBTR).

In the JK biobank validation cohort, we observed distinct associations between the epithelial cell states and clinicopathological variables. S04 abundance was strongly correlated with Immunescore (rho 0.74, *p* < 0.001), ProliferativeIndex (rho 0.61, *p* < 0.001), and histological grade (rho 0.44, *p* < 0.001) and inversely correlated with ESR1 (rho -0.31, *p* < 0.001), Figure S3. The S05 epithelial cell state correlated with ESR1 (rho 0.47, *p* < 0.001), ERBB2 (rho 0.39, *p* < 0.001), and age (rho 0.29, *p* < 0.001) and was inversely correlated with Immunescore (rho -0.21, *p* < 0.001), ProliferativeIndex (rho = -0.29, *p* < 0.001), and histological grade (rho -0.20, *p* < 0.001). Similar contrasting immunomodulatory effects of the S04 and S05 epithelial cell states were seen in the ER-positive subset in univariable (p_interaction_ 0.048 and 0.046, respectively) and multivariable (p_interaction_ 0.13 and 0.011, respectively) analysis. The findings remained robust when analyzing the whole cohort, including ER-negative tumors (univariable; p_interaction_ 0.009 and 0.001, respectively, multivariable; p_interaction_ 0.043 and <0.001, respectively). These findings suggest that the S04 epithelial cell state represents a highly proliferative, immune-permissive tumor trait, whereas S05 reflects tumors with ineffective or absent immune infiltration.

### Associations Between the S04 and S05 Epithelial Cell States and the Tumor Microenvironment

**Fig. 3:**
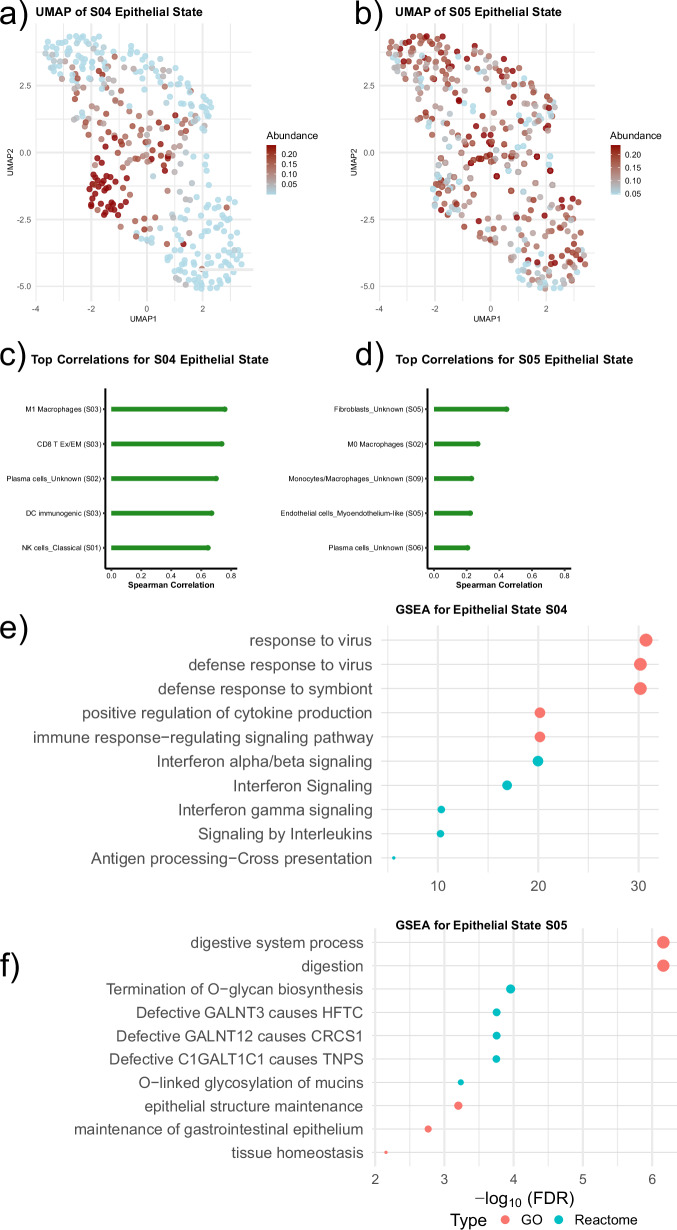
Immune and molecular correlates of S04 and S05 epithelial cell states in ER-positive breast cancers of the JK biobank cohort. **a**, **b** UMAP plots visualizing ER-positive tumor samples based on EcoTyper-inferred tumor microenvironment composition, colored by relative abundance of the S04 (**a**) and S05 (**b**) epithelial cell states. Tumors with high S04 abundance cluster together, indicating a coordinated immune-infiltrated microenvironment, while S05 abundance shows greater heterogeneity. **c**, **d** Top Spearman correlations between epithelial states and EcoTyper-defined immune/stromal cell states in ER-positive tumors. S04 (**c**) is positively associated with M1-like macrophages, exhausted/effector (Ex/EM) memory CD8⁺ T cells, plasma cells, and immunogenic dendritic cells (DC), suggesting an immune-permissive phenotype. S05 (**d**) correlates with fibroblasts and myoepithelial-like endothelial cells consistent with an immune-excluded/immune-evasive phenotype. **e** Gene set enrichment analysis (GSEA) of differentially expressed genes in the S04 cell state shows enrichment for immune-related pathways, including type I and II interferon signaling, antigen presentation, cytokine signaling, and antiviral defense responses. **f** GSEA for the S05 epithelial state reveals enrichment of pathways related to O-glycan biosynthesis, mucin biology, epithelial homeostasis, and gastrointestinal tissue maintenance, consistent with an epithelial-differentiated but immune-evasive phenotype. Note: GSEA analyses are based on the original EcoTyper-defined gene sets for the respective epithelial cell states and not restricted to differentially expressed genes from our ER-positive cohort.

To better characterize how the epithelial S04 and S05 cell states influence the TME in ER-positive tumors, we investigated their associations with EcoTyper-defined immune and stromal cell states. S04 abundance was positively correlated with multiple pro-inflammatory immune states, including M1-like macrophages (Macrophages S03), exhausted/effector memory CD8 + T cells (CD8 T cells S03), and plasma cells (Plasma cells S02). UMAP projections demonstrated a clustered distribution of the epithelial cell S04 abundance score suggesting coordinated changes in the broader TME, Fig. 3. In contrast, the S05 epithelial cell state was associated with a fibroblast state of unknown function (S05) and showed weaker associations with immune cell states, Figure S4. UMAP analysis demonstrated a heterogeneous distribution of S05 abundance, indicating greater variability in its associated TME. The S04 epithelial cell state was most strongly associated with the original CE9 ecotype, followed by CE10, while the S05 state showed a more heterogeneous ecotype distribution, consistent with UMAP findings, Figure S5. Further analysis of the fibroblast S05 cell state showed enrichment for cilium-related processes and endocrine therapy resistance (Figure S6).

### Gene Set Enrichment for S04 and S05 Epithelial Cell States

Enrichment analysis of constituent genes of the S04 epithelial state revealed significant overrepresentation of immune-related processes spanning both innate and adaptive immune responses. Pathways included antiviral defense response (e.g., “response to virus”, “defense response to virus”), interferon signaling (type I and II), antigen processing and presentation, and cytokine/chemokine production/interactions, Supplementary Data  S7, supporting the S04 epithelial cell state as an immune-permissive tumor-intrinsic trait in ER-positive tumors.

In contrast, S05 was enriched for processes related to glycosylation-/mucin-related processes (e.g., “digestion”, “maintenance of gastrointestinal epithelium”, “Termination of O-glycan biosynthesis”, “O-linked glycosylation of mucins”) involving protein nodes related to glycosylation and mucin biology (MUC1, MUC5B, MUC13, ST6GAL1, GCNT3)^21–26^, Supplementary Data S8. Pathways related to epithelial integrity and homeostasis (e.g. “epithelial structure and maintenance”, “tissue homeostasis”, “IRE1-mediated unfolded protein response”, and “response to endoplasmic reticulum stress”) were also enriched involving protein nodes such as CLDN3 and CLDN7^27,28^. Additional top enriched protein nodes included MAPK3, ERBB2, and AGR2.

### Methylation Analysis of Differentially Methylated Genes for the S04 and S05 Epithelial Cell States

To further explore the mechanisms underlying the different epithelial cell states, we analyzed differential methylation patterns in ER-positive tumors for the S04 and S05 traits, respectively, while adjusting for the proportion of stromal and immune cells and filtering for sites predicted to be differentially methylated within the epithelial compartment (see Methods section). Differentially hypomethylated sites of the S04 cell state demonstrated enrichment for interferon signaling (e.g., *STAT1, MX1, NLRC5, DDX60, IRF1*, and *IRF8*^29–32^), Poly-ADP-Ribose polymerases (e.g., *PSMB8, PARP9, PARP12, PARP14*), and antigen presentation (e.g., *TAP1, B2M*, and *MX1*), Figure S7, Supplementary Data S9, Supplementary Data S10. In contrast, hypermethylated sites for the S04 cell state were enriched primarily for the PI3K pathway, Supplementary Data S11. The S05 cell state, in contrast, demonstrated hypomethylation of gene sites related to metabolic pathways (e.g., *OGDH, NANS, SLC37A1*, and *SLC22A18*^33–37^), and adhesion molecules (e.g., *ITGB4, CEACAM6*), Supplementary Data S12. Top protein nodes included ERBB2, ITG4, and PXN. Both cell states demonstrated hypermethylation in EZH2, the catalytic subunit of PRC2, but the S04 cell state involved immune-related genes unlike the S05 cell state which involved genes primarily related to metabolism and extracellular matrix modulations (e.g., *CHST8, DUOXA2, ADCY5*^38–40^).

## Discussion

The role of the immune system in ER-positive breast tumors is complex and incompletely understood. This study integrates immune infiltration assessments, methylation analysis, and profiling of tumor-intrinsic epithelial cell states to identify tumor-intrinsic traits with distinct epigenetic/genetic profiles and effects on the tumor immune microenvironment. These findings underscore the context-dependent nature of the immune infiltrate’s abundance and function, and provide a framework for stratifying patients with ER-positive tumors based on their immune biology, which may inform treatment decisions and guide efforts aimed to facilitate an antitumoral immune response.

ER-positive tumors are generally considered immune-cold, partly due to estrogen-mediated suppression of immune-related pathways^41^. Prior studies on the prognostic effects of immune infiltration in ER-positive tumors have yielded conflicting results^5,42,43^, likely due to a failure to account for confounders or tumor-intrinsic factors that modulate immune responsiveness. However, emerging evidence suggests that a subset of ER-positive tumors may be immunologically active and responsive to checkpoint blockade^10^. Recent phase 3 trials have demonstrated improved pathological complete response rates with the addition of immunotherapy to neoadjuvant chemotherapy in high-risk ER-positive/HER2-negative breast cancer—specifically with pembrolizumab in the KEYNOTE-756 trial^44^ and nivolumab in NCT04109066^45^. In the latter, the benefit was most pronounced in tumors with high TILs or PD-L1 expression^45^. These findings underscore the need to identify biological features that distinguish immune-responsive from immune-inert tumor traits in ER-positive tumors. Our findings align with the notion of aggressive tumor characteristics, as captured by proliferation, being permissive of a favorable immune response. Furthermore, they offer a potential refinement of current high-risk definitions by incorporating epithelial state profiling and immune signatures, which may better capture immunotherapy-responsive biology than proliferation, histological grade, or TILs/PD-L1 alone.

Leveraging pan-cancer epithelial cell states^18^, we identified the S04 epithelial cell state as a proliferation-associated epithelial cell state in ER-positive tumors associated with broad TME changes. This involved pro-inflammatory cell states suggesting a coordinated immune response involving both innate and adaptive subsets. M1-like macrophages showed the strongest correlation with S04 abundance, consistent with recent findings implicating macrophages in shaping immune-permissive niches^46^. Gene set enrichment and methylation analyses further supported this interpretation, highlighting immune activation pathways and hypomethylation of both innate and adaptive immune signaling genes. We also observed hypermethylation of PI3K pathway components, known to have immunosuppressive effects in breast cancer^47,48^, reinforcing the rationale for clinical trials combining immunotherapy with PI3K inhibition (NCT04208178). Our findings provide a biological link between tumor proliferation and immune responsiveness in ER-positive breast cancer, suggesting that epithelial phenotyping could improve patient selection for immune-targeted treatments.

Conversely, the S05 epithelial cell state lacked strong correlations with most immune cell states and demonstrated a more heterogeneous distribution across the TME. S05 abundance correlated with ESR1 expression, consistent with previous studies linking the level of ER expression with immune competence and responsiveness to checkpoint blockade^49,50^. Gene set enrichment analysis revealed enrichment for mucin-related pathways, consistent with prior reports linking mucinous tumors with immune evasion^51^ and mucin-related pathways as potential immune evasion mechanisms^52–56^. S05 epithelial abundance correlated with ERBB2 expression, despite HER2-positive tumors generally being regarded as more likely to be immune-infiltrated. One possible explanation is that HER2-targeted therapies, which were not administered in the analyzed cohort, are known to exert indirect immune-mediated effects^57–59^; their absence may have made these tumors less immune-responsive. Alternatively, although HER2-positive tumors may exhibit immune infiltration, this does not necessarily translate into effective anti-tumoral immunity, as suggested by the limited benefit observed in immunotherapy trials in clinical trials^60^. Furthermore, S05 abundance also correlated with abundance of the previously incompletely characterized fibroblast S05 cell state, whose transcriptional profile was enriched for cilium-related processes and genes linked to endocrine therapy resistance. Emerging evidence suggests that primary cilia drive the differentiation of adipose progenitors to fibroblasts which, in turn, increase ERBB ligand-receptor interactions, contributing to a TME that supports estrogen signaling-independent growth and resistance to therapy^61,62^. Given the association with immune dysfunction, the S05 epithelial cell state may serve as a biomarker to further distinguish immune-permissive from immune-evasive traits in ER-positive breast cancer.

Current guidelines emphasize the importance of a radiotherapy (RT) boost after whole-breast irradiation in high-risk patients, such as those aged <50 or aged 50-65 with histological grade 3 tumors, given the significant benefit in these patients^19,20^. While omission may be considered in patients at higher risk for normal tissue toxicity^41^, there are no guidelines for de-escalation based on tumor biology. Our findings indicate that immune biomarkers can fill this gap. Specifically, patients with high Immunescore and high ProliferativeIndex had outcomes comparable to those of lower-risk patients, suggesting that a robust immune response may partially compensate for aggressive tumor biology and reduce the need for intensified local therapy. While proliferation appears to be a key prognostic factor in older low-risk patients, the immune response likely holds greater significance in younger patients or those with more aggressive biology. Together, our results support the integration of immune biomarkers, alongside proliferation and epithelial profiling, into future trials exploring biologically guided de-escalation of RT. Beyond RT, future studies should also explore the predictive impact of the immune infiltrate on the benefit from endocrine therapy.

Several limitations should be acknowledged for the current study. Although our cohort predates modern systemic treatment such as HER2-targeted treatment, immunotherapy, and CDK4/6 inhibitors, our results align with data including modern treatment^10^. We show that immune biomarkers can be used to refine RT decisions, but future studies should investigate potential synergies with modern systemic treatment^63^. We used multivariable models to account for potential confounders, but the retrospective non-randomized design and imputation of missing data may still result in residual bias. We did not perform pairwise matching between the RT groups to avoid loss of statistical power in our imbalanced cohort and although propensity score adjustment was used to address confounding, residual imbalances cannot be excluded. Assessment of TILs remains the most reliable measure of global immune infiltration, and our reliance on gene expression-based inference represents another limitation. While IHC-validation of the immune signature was not performed in this cohort, it has been validated previously and shown to outperform other gene expression-based deconvolutional tools, supporting its reliability as a surrogate measure of immune infiltration^9^. In addition, a minority of samples analyzed (*n* = 10) were derived from FFPE tissue collected in the 1990s and expectedly showed lower RNA quality. Nevertheless, these samples were retained for analysis, as post-correction assessments did not indicate substantial batch-related bias. Another limitation is that HER2 status was assessed using gene expression instead of IHC although the agreement between gene expression-based and IHC-based methods tends to be high^64,65^. The ligand-based assay used to determine ER expression^66^ showed excellent concordance with PAM50-like subtyping, but it would have been preferred to use IHC instead. Most contemporary guidelines rely on IHC with a ≥ 1% nuclear staining cut-off to define ER-positivity and prior studies suggest that a 25 fmol/mg ligand-based assay threshold roughly corresponds to >10% IHC staining^67^. This limits direct comparison to modern data sets. However, our classification likely reflects tumors with more robust ER expression, making the results particularly applicable to clinically relevant ER-positive disease. Given these limitations, our findings should be validated in prospective trials. Nevertheless, our results were consistent across subgroups and cohorts and align with prior literature which supports their robustness.

In conclusion, we identified tumor-intrinsic traits related to proliferation that shape the tumor microenvironment in ER-positive tumors. We characterized tumors enriched for immune-related processes on a genetic and epigenetic level, supporting the concept of an immunotherapy-responsive ER-positive subset. Our findings establish a framework for integrating tumor-intrinsic epithelial states with immune profiling, which can be applied in future studies investigating immunomodulatory treatments.

## Methods

### Study population

M0 tumors from breast cancer patients younger than 80 years operated with breast-conserving surgery and diagnosed in Western Sweden between 1989 and 1999 were identified from the Jubilee Clinic biobank (JK biobank), Fig. 1. Medical records were reviewed to collect clinical data, including tumor size, lymph node status, histological grade, and treatment details such as radiotherapy (RT), endocrine therapy, and chemotherapy. Outcome information regarding ipsilateral breast tumor recurrence (IBTR), regional recurrence, distant metastasis, breast cancer-specific death, and overall survival was collected. ER and progesterone receptor (PgR) status were assessed using a ligand-based enzyme immunoassay at the time of diagnosis with a threshold of ≥25 fmol/mg protein used to define positivity according to contemporary guidelines^66,68^. While current guidelines are based on IHC, prior studies suggest that this threshold would roughly correspond to >10% nuclear staining by IHC^67^. A total of 31 tumors overlapping with the SweBCG91RT cohort were excluded to avoid redundancy with prior analyses of the interaction between Immunescore and ProliferativeIndex^9^. HER2 status was approximated using gene expression using the genefu R package. Breast cancer subtypes were defined based on the St Gallen 2013 definition^69^ and further refined according to studies from Maisonneuve^70^ and Ehinger^71^, using ER status, HER2 status, histological grade (imputed in 20.6% of cases, see below for further details), and PgR status to categorize tumors into Luminal A (ER-positive, HER2-negative, histological grade 1 or histological grade 2 and PgR-positive), Luminal B (ER-positive, HER2-negative, histological grade 3 or histological grade 2 and PgR-negative), HER2-positive (HER2-positive), and non-luminal (ER-negative, any histological grade and PgR status) subtypes. Immunescore and ProliferativeIndex were calculated for each sample, as previously described^9^.

Tumor samples were obtained pre-treatment (systemic or radiation therapy) and no patients received neoadjuvant therapy. Total RNA was extracted from either fresh-frozen tumor samples or formalin-fixed, paraffin-embedded (FFPE) tissue sections when fresh-frozen samples were unavailable. RNA integrity was assessed using the Agilent Tapestation 4200. RNA concentration was measured using the Invitrogen Qubit RNA HS Assay Kit (ThermoFisher Scientific). Fresh-frozen samples with an RNA Integrity Number (RIN) between 2.0 and 9.0 were included for downstream analyses, while FFPE samples had RIN values ranging between 1.5 and 2.5. Library preparation was conducted at the Science for Life Laboratory (National Genomics Infrastructure, Uppsala). Illumina TruSeq strand-specific RNA libraries were generated, incorporating ribosomal RNA depletion using the RiboZero human kit. Paired-end sequencing (125 bp) was performed on an Illumina HiSeq2000 platform, yielding approximately 15 to 30 million aligned reads per sample (median: 22.5 million aligned reads). Read alignment and computations were carried out using resources provided by the Swedish National Infrastructure for Computing (SNIC) through UPPMAX. Reads were aligned using STAR aligner and gene expression levels were quantified using RSEM. Data were normalized using variance stabilizing transformation (VST) and batch effects were corrected using the removeBatchEffect function from the limma package. A total of 11 FFPE samples were available, 10 of which had matching clinical data and were included in downstream analysis. Data quality was assessed by quality control metrics including library complexity, entropy, gene detection rate, and principal component analysis. FFPE-derived samples (batch 3) showed reduced library size and detected genes but did not form distinct clusters in the PCA space post-batch correction, Figure S8. Therefore, all samples were retained to maximize statistical power. A total of 428 samples, of which 344 (355 after imputations, see below) were ER-positive, had available clinical and molecular information.

Methylation data were generated using the Illumina Infinium Methylation EPIC array. A total of 380 tumors had available DNA tissue for methylation analysis, of which 270 were ER-positive and included in analysis. Bisulfite conversion was performed using the EZ DNA Methylation Kit (Zymo Research, D5004). Beta values were used as measures of methylation, ranging from 0 (no methylation) to 1 (full methylation). GenomeStudio (v2011.1) was used for methylation data processing, and four CEPH/NA12878 control samples were included to monitor batch reproducibility (Pearson r = 0.994). A sample was considered acceptable if its probe call rate exceeded 98% at a detection *p*-value of 0.01, corresponding to at least 848,599 approved CpG loci.

This retrospective study was conducted in accordance with the Declaration of Helsinki and received approval from the regional ethics committee of Western Sweden (approval numbers 162-02, 768-14, T530-16, and 2023-03031-02). Informed oral consent for tissue biobanking was obtained at the time of diagnosis from all patients. The ethics committee determined that the original informed oral consent was appropriate for the present study. The REMARK guidelines were followed when performing the study.

### Public Data sets

Publicly available breast cancer data sets were downloaded using the MetaGxBreast R package^72^. In addition, the Servant^73^ data set was used. A total of 21 cohorts with outcome data were selected as a training set, and endpoints were selected in the following order of priority: any recurrence, distant metastasis, local recurrence and overall survival within 10 years of follow-up, Supplementary Data S13. ER-positive tumors were selected for analysis and Cox regression models were used to evaluate the interaction between Immunescore and epithelial cell states defined by EcoTyper (see below) to identify tumor-intrinsic biology influencing immune responsiveness or immune-evasion. This was done in the training set with ProliferativeIndex as covariate to adjust for proliferation. Epithelial cell states with an adjusted *P* value (Benjamini-Hochberg) < 0.05 were validated in the JK biobank validation cohort with adjustment for the same covariates as described in the statistical methods section (see below).

### EcoTyper and Enrichment Analysis

EcoTyper^18^ was used to profile six distinct epithelial cell states (S01-S06) across the breast cancer samples and their association with immune responsiveness, defined as tumor-intrinsic characteristics permissive of an active immune infiltrate linked to improved clinical outcomes. This concept expands beyond the mere presence of immune cells and aims to also capture their functional activity within the TME. For all analyses, we used continuous abundance scores rather than discrete assignments to preserve statistical power and avoid information loss. As such, tumors were not strictly “assigned” to a specific cell state.

To explore how the identified epithelial cell states of primary interest (S04 and S05 states) influence the surrounding TME, we performed a series of complementary analyses in the ER-positive subset. First, we calculated Spearman correlation coefficients between the continuous abundance scores of the S04 and S05 epithelial cell states and all other EcoTyper-defined immune and stromal cell states. The top associations were visualized using lollipop plots. UMAP dimensionality reduction was performed using the abundance matrix of all immune and stromal cell states, excluding the S04 and S05 epithelial cell states. Abundance scores for S04 and S05 were then overlaid on the UMAP projection to assess whether these states were associated with global shifts in TME composition.

Pathway enrichment analyses were performed for the S04 and S05 cell states using their respective gene sets using clusterProfiler^74^ (Gene Ontology), ReactomePA^75^ (Reactome pathways), STRINGdb^76^ (protein nodes), and enrichR^77^ (transcription factor motifs). The top 10 enriched results from each database were included in the final tables. Bubble plots were generated for Gene Ontology and Reactome results. Protein node and TF motif enrichments were excluded from the plots due to their low significance and broad overlap/low specificity, respectively. Given the strong correlation between the epithelial S05 and the unknown fibroblast S05 cell states, gene set enrichment analysis was also performed for the fibroblast S05 state to further investigate potential stromal contributions to immune modulation in tumors enriched for the epithelial S05 state. All *p* values for enrichment analyses were adjusted using FDR.

To explore the potential role of epigenetic modifications in immune regulation, methylation patterns were analyzed for the S04 and S05 epithelial cell states in ER-positive tumors. Differentially methylated CpG sites were identified using limma, adjusting for epithelial and stromal/immune proportions using EpiDISH^78^. The results were intersected with methylation sites significantly altered within epithelial compartments (FDR < 0.05) as determined by TOAST^79^ to improve epithelial specificity. Enrichment analyses of the resulting gene sets were conducted using pathway tools described above. Volcano plots were generated to visualize the results.

### Statistical methods

The primary endpoint was time to IBTR as first event within 10 years. Regional recurrence, distant recurrence, and any death were considered competing risks to IBTR. IBTR was chosen as endpoint given its direct relevance to RT. Time-to-event outcomes were modeled using Cox proportional hazards regression. Likelihood ratio tests were performed to assess the interaction between Immunescore and ProliferativeIndex on IBTR risk. Patients were censored at 10 years from diagnosis or at the latest known follow-up. Hazard ratios (HRs) and 95% confidence intervals (CIs) were calculated. Covariates were tested in univariable analysis and significant variables or variables deemed biologically relevant were kept in multivariable analysis. Covariates tested were pathological subtype, RT, systemic treatment, radical surgery, tumor size, histological grade, number of positive lymph nodes, PgR, and age. Given the overlap between subtype and histological grade, only subtype was retained in the final analysis despite both variables being significant in univariable analysis. The proportional hazards assumption was tested using the Grambsch-Therneau test (cox.zph function in R) and fulfilled for all variables in the ER-positive subset analyses and for all variables except ProliferativeIndex when analyzing all patients. The hazard ratio for ProliferativeIndex in analysis of the whole cohort should thus be regarded as an average over the 10-year follow-up time. Missing values for continuous variables were imputed with the mice package^80^ using the Multivariate Imputation by Chained Equations (MICE) framework. A total of 10 iterations were run to ensure convergence, and a single imputed data set was used for downstream analysis. Predictive mean matching (PMM) was applied for continuous variables while logistic regression was used for binary or categorical variables, Supplementary Data S14. To improve imputation accuracy, the model included clinical covariates used in survival modeling as well as s-phase fraction, year of diagnosis, and key biological scores such as Immunescore and ProliferativeIndex.

Flexible parametrics models were created to model the predicted risk of IBTR depending on RT treatment across strata defined by the median of tumors within the lowest quartile, overall median, and median of tumors within the highest quartile of Immunescore and ProliferativeIndex. Separate models were fitted for irradiated and unirradiated patients and backwards selection was performed starting with the same covariates as above (full models available in the supplement). In addition, the curves for irradiated and unirradiated patients were matched by propensity scores to adjust for confounders associated with RT. Propensity scores were calculated using a logistic regression model with backwards selection of the variables age, tumor size, radical surgery, systemic treatment, lymph node status, and diagnosis date in the initial model. Of these, age and radical surgery remained significant and were retained to fit the final propensity score model, Supplementary Data S14.

Cumulative incidence plots were created according to the method of Fine and Gray^81^. Curves stratified by median Immunescore were generated for selected high-risk groups who, based on current guidelines, may be recommended RT boost. These were (1) patients <50 years treated with RT, systemic therapy, and radical breast-conserving surgery and (2) patients aged 50–65 with histological grade III treated with the same combination. The differences between groups were assessed using Gray’s test.

## Supplementary information

Supplement 6-12

Supplementary DataS1

Supplementary DataS2

Supplementary DataS3

Supplementary DataS4

Supplementary DataS5

Supplementary DataS6

Supplementary DataS7

Supplementary DataS8

Supplementary DataS9

Supplementary DataS10

Supplementary DataS11

Supplementary DataS12

Supplementary DataS13

Supplementary DataS14

## Data Availability

The data sets generated and analyzed during the current study have been deposited to the GEO (GSE298750) and SRA (BioProject: 1280695) repositories.

## Code availability

Custom code used in this study is available upon reasonable request from the corresponding author.

## References

[1] Amens, J. N., Bahçecioglu, G. & Zorlutuna, P. Immune system effects on breast cancer. *Cell Mol. Bioeng.* **14**, 279–292 (2021). 34295441 10.1007/s12195-021-00679-8 PMC8280260

[2] El Bairi, K. et al. The tale of TILs in breast cancer: a report from The International Immuno-Oncology Biomarker Working Group. *NPJ Breast Cancer* **7**, 150 (2021). 34853355 10.1038/s41523-021-00346-1 PMC8636568

[3] Fujimoto, Y. et al. Prognostic significance of tumor-infiltrating lymphocytes may differ depending on Ki67 expression levels in estrogen receptor-positive/HER2-negative operated breast cancers. *Breast Cancer* **26**, 738–747 (2019). 31098866 10.1007/s12282-019-00977-0

[4] Lundgren, C., et al. Tumour-infiltrating lymphocytes as a prognostic and tamoxifen predictive marker in premenopausal breast cancer: data from a randomised trial with long-term follow-up. *Breast Cancer Res.* **22**, 140 (2020). 33357231 10.1186/s13058-020-01364-w PMC7758933

[5] He, J. et al. Prognostic value of tumour-infiltrating lymphocytes based on the evaluation of frequency in patients with oestrogen receptor-positive breast cancer. *Eur. J. Cancer* **154**, 217–226 (2021). 34293665 10.1016/j.ejca.2021.06.011

[6] Denkert, C. et al. Tumour-infiltrating lymphocytes and prognosis in different subtypes of breast cancer: a pooled analysis of 3771 patients treated with neoadjuvant therapy. *Lancet Oncol.* **19**, 40–50 (2018). 29233559 10.1016/S1470-2045(17)30904-X

[7] Sobral-Leite, M. et al. Cancer-immune interactions in ER-positive breast cancers: PI3K pathway alterations and tumor-infiltrating lymphocytes. *Breast Cancer Res.* **21**, 90 (2019). 31391067 10.1186/s13058-019-1176-2 PMC6686400

[8] McGuinness, C. & Britt, K. L. Estrogen receptor regulation of the immune microenvironment in breast cancer. *J. Steroid Biochem Mol. Biol.* **240**, 106517 (2024). 38555985 10.1016/j.jsbmb.2024.106517

[9] Stenmark Tullberg, A. et al. Integrating tumor-intrinsic and immunologic factors to identify immunogenic breast cancers from a low-risk cohort: results from the randomized SweBCG91RT trial. *Clin. Cancer Res.* **29**, 1783–1793 (2023). 37071498 10.1158/1078-0432.CCR-22-2746 PMC10150244

[10] Rios-Hoyo, A. et al. Neoadjuvant chemotherapy and immunotherapy for estrogen receptor-positive human epidermal growth factor 2-negative breast cancer. *J. Clin. Oncol.* **42**, 2632–2636 (2024). 38593393 10.1200/JCO.23.02614

[11] Nanda, R. et al. Effect of pembrolizumab plus neoadjuvant chemotherapy on pathologic complete response in women with early-stage breast cancer: an analysis of the ongoing phase 2 adaptively randomized I-SPY2 trial. *JAMA Oncol.* **6**, 676–684 (2020). 32053137 10.1001/jamaoncol.2019.6650 PMC7058271

[12] Kunkler, I. H. et al. Breast-conserving surgery with or without irradiation in women aged 65 years or older with early breast cancer (PRIME II): a randomised controlled trial. *Lancet Oncol.* **16**, 266–273 (2015). 25637340 10.1016/S1470-2045(14)71221-5

[13] Sjöström, M. et al. Development and Validation of a Genomic Profile for the Omission of Local Adjuvant Radiation in Breast Cancer. *J. Clin. Oncol.* **41**, 1533–1540 (2023). 36599119 10.1200/JCO.22.00655 PMC10022846

[14] Sjöström, M. et al. Clinicogenomic radiotherapy classifier predicting the need for intensified locoregional treatment after breast-conserving surgery for early-stage breast cancer. *J. Clin. Oncol.* **37**, 3340–3349 (2019). 31618132 10.1200/JCO.19.00761 PMC6901281

[15] Karlsson, P. et al. Validation of a breast cancer assay for radiotherapy omission: an individual participant data meta-analysis. *JNCI: Journal of the National Cancer Institute*, 2024: p. djae262. 10.1093/jnci/djae262 PMC11884857 39423142

[16] Stenmark Tullberg, A. et al. Combining histological grade, TILs, and the PD-1/PD-L1 pathway to identify immunogenic tumors and de-escalate radiotherapy in early breast cancer: a secondary analysis of a randomized clinical trial. *J. Immunother Cancer*. **11**, (2023). 10.1136/jitc-2022-006618 PMC10201214 37208129

[17] Stenmark Tullberg, A. et al. Immune infiltrate in the primary tumor predicts effect of adjuvant radiotherapy in breast cancer; results from the randomized SweBCG91RT Trial. *Clin. Cancer Res.* **27**, 749–758 (2021). 33148672 10.1158/1078-0432.CCR-20-3299

[18] Luca, B. A. et al. Atlas of clinically distinct cell states and ecosystems across human solid tumors. *Cell* **184**, 5482–5496.e28 (2021). 34597583 10.1016/j.cell.2021.09.014 PMC8526411

[19] Antonini, N. et al. Effect of age and radiation dose on local control after breast conserving treatment: EORTC trial 22881-10882. *Radiother. Oncol.* **82**, 265–271 (2007). 17126434 10.1016/j.radonc.2006.09.014

[20] Gradishar, W. J. et al. Breast Cancer, Version 3.2022, NCCN clinical practice guidelines in oncology. *J. Natl Compr. Canc Netw.* **20**, 691–722 (2022). 35714673 10.6004/jnccn.2022.0030

[21] Cascio, S. & Finn, O. J. Intra- and extra-cellular events related to altered glycosylation of MUC1 promote chronic inflammation, tumor progression, invasion, and metastasis. *Biomolecules* **6** (2016). 10.3390/biom6040039 PMC5197949 27754373

[22] Apostolopoulos, V., Stojanovska, L. & Gargosky, S. E. MUC1 (CD227): a multi-tasked molecule. *Cell Mol. Life Sci.* **72**, 4475–4500 (2015). 26294353 10.1007/s00018-015-2014-z PMC11113675

[23] Beatson, R. et al. The mucin MUC1 modulates the tumor immunological microenvironment through engagement of the lectin Siglec-9. *Nat. Immunol.* **17**, 1273–1281 (2016). 27595232 10.1038/ni.3552 PMC5257269

[24] Beckwith, D. M. & Cudic, M. Tumor-associated O-glycans of MUC1: carriers of the glyco-code and targets for cancer vaccine design. *Semin Immunol.* **47**, 101389 (2020). 31926647 10.1016/j.smim.2020.101389 PMC7160022

[25] Valque, H. et al. MUC5B leads to aggressive behavior of breast cancer MCF7 cells. *PLoS ONE* **7**, e46699 (2012). 23056409 10.1371/journal.pone.0046699 PMC3462796

[26] Park, J. H. et al. Critical roles of mucin 1 glycosylation by transactivated polypeptide N-acetylgalactosaminyltransferase 6 in mammary carcinogenesis. *Cancer Res.* **70**, 2759–2769 (2010). 20215525 10.1158/0008-5472.CAN-09-3911

[27] Kominsky, S. L. et al. Loss of the tight junction protein claudin-7 correlates with histological grade in both ductal carcinoma in situ and invasive ductal carcinoma of the breast. *Oncogene* **22**, 2021–2033 (2003). 12673207 10.1038/sj.onc.1206199

[28] Lin, X. et al. Regulation of the epithelial-mesenchymal transition by Claudin-3 and Claudin-4. *PLoS ONE* **8**, e67496 (2013). 23805314 10.1371/journal.pone.0067496 PMC3689737

[29] Mostafavi, S. et al. Parsing the Interferon Transcriptional Network and Its Disease Associations. *Cell* **164**, 564–578 (2016). 26824662 10.1016/j.cell.2015.12.032 PMC4743492

[30] Mogensen, T. H. IRF and STAT transcription factors - from Basic biology to roles in infection, protective immunity, and primary immunodeficiencies. *Front Immunol.* **9**, 3047 (2018). 30671054 10.3389/fimmu.2018.03047 PMC6331453

[31] Forero, A. et al. Differential activation of the transcription factor IRF1 underlies the distinct immune responses elicited by Type I and Type III interferons. *Immunity* **51**, 451–464.e6 (2019). 31471108 10.1016/j.immuni.2019.07.007 PMC7447158

[32] Langlais, D., Barreiro, L. B. & Gros, P. The macrophage IRF8/IRF1 regulome is required for protection against infections and is associated with chronic inflammation. *J. Exp. Med.* **213**, 585–603 (2016). 27001747 10.1084/jem.20151764 PMC4821649

[33] Bunik, V. I. & Fernie, A. R. Metabolic control exerted by the 2-oxoglutarate dehydrogenase reaction: a cross-kingdom comparison of the crossroad between energy production and nitrogen assimilation. *Biochem. J.* **422**, 405–421 (2009). 19698086 10.1042/BJ20090722

[34] Shtraizent, N. et al. MPI depletion enhances O-GlcNAcylation of p53 and suppresses the Warburg effect. *Elife* **6** (2017). 10.7554/eLife.22477 PMC5495572 28644127

[35] van Karnebeek, C. D. et al. NANS-mediated synthesis of sialic acid is required for brain and skeletal development. *Nat. Genet.* **48**, 777–784 (2016). 27213289 10.1038/ng.3578

[36] Nigam, S. K. The SLC22 transporter family: a paradigm for the impact of drug transporters on metabolic pathways, signaling, and disease. *Annu Rev. Pharm. Toxicol.* **58**, 663–687 (2018). 10.1146/annurev-pharmtox-010617-052713 PMC6225997 29309257

[37] Iacopetta, D. et al. SLC37A1 gene expression is up-regulated by epidermal growth factor in breast cancer cells. *Breast Cancer Res. Treat.* **122**, 755–764 (2010). 19894109 10.1007/s10549-009-0620-x

[38] Chou, W.-C. et al. Genetic insights into carbohydrate sulfotransferase 8 and its impact on the immunotherapy efficacy of cancer. *Cell Rep.* **43**, 113641 (2024). 38165805 10.1016/j.celrep.2023.113641

[39] Guo, R. et al. Targeting adenylate cyclase family: new concept of targeted cancer therapy. *Front Oncol.* **12**, 829212 (2022). 35832555 10.3389/fonc.2022.829212 PMC9271773

[40] Faria, C. C. & Fortunato, R. S. The role of dual oxidases in physiology and cancer. *Genet Mol. Biol.* **43**, e20190096 (2020). 32453337 10.1590/1678-4685/GMB-2019-0096 PMC7265977

[41] Smith, B. D. et al. Radiation therapy for the whole breast: Executive summary of an American Society for Radiation Oncology (ASTRO) evidence-based guideline. Pract. *Radiat. Oncol.* **8**, 145–152 (2018). 10.1016/j.prro.2018.01.012 29545124

[42] Heindl, A. et al. Relevance of spatial heterogeneity of immune infiltration for predicting risk of recurrence after endocrine therapy of ER+ breast cancer. *J. Natl. Cancer Inst*. **110** (2018). 10.1093/jnci/djx137 PMC6298573 28859291

[43] Bense, R. D. et al. Relevance of Tumor-Infiltrating Immune Cell Composition and Functionality for Disease Outcome in Breast Cancer. *J. Natl. Cancer Inst*. **109** (2017). 10.1093/jnci/djw192 PMC6284248 27737921

[44] Cardoso, F. et al. Pembrolizumab and chemotherapy in high-risk, early-stage, ER + /HER2− breast cancer: a randomized phase 3 trial. *Nat. Med.* **31**, 442–448 (2025). 39838117 10.1038/s41591-024-03415-7 PMC11835712

[45] Loi, S. et al. Neoadjuvant nivolumab and chemotherapy in early estrogen receptor-positive breast cancer: a randomized phase 3 trial. *Nat. Med.* **31**, 433–441 (2025). 39838118 10.1038/s41591-024-03414-8 PMC11835735

[46] Onkar, S. et al. Immune landscape in invasive ductal and lobular breast cancer reveals a divergent macrophage-driven microenvironment. *Nat. Cancer* **4**, 516–534 (2023). 36927792 10.1038/s43018-023-00527-w PMC11194444

[47] Qin, H. et al. The impact of PI3K inhibitors on breast cancer cell and its tumor microenvironment. *PeerJ* **6**, e5092 (2018). 29942710 10.7717/peerj.5092 PMC6014315

[48] Sai, J. et al. PI3K Inhibition Reduces Mammary Tumor Growth and Facilitates Antitumor Immunity and Anti-PD1 Responses. *Clin. Cancer Res.* **23**, 3371–3384 (2017). 28003307 10.1158/1078-0432.CCR-16-2142 PMC5479746

[49] Voorwerk, L. et al. Immune landscape of breast tumors with low and intermediate estrogen receptor expression. *NPJ Breast Cancer* **9**, 39 (2023). 37179445 10.1038/s41523-023-00543-0 PMC10182974

[50] Massa, D. et al. Immune and gene-expression profiling in estrogen receptor low and negative early breast cancer. *J. Natl Cancer Inst.* **116**, 1914–1927 (2024). 39083015 10.1093/jnci/djae178 PMC11630536

[51] Nguyen, B. et al. Genomic, Transcriptomic, Epigenetic, and Immune Profiling of Mucinous Breast Cancer. *J. Natl Cancer Inst.* **111**, 742–746 (2019). 30789657 10.1093/jnci/djz023

[52] Lv, W. et al. Analysis of Tumor Glycosylation Characteristics and Implications for Immune Checkpoint Inhibitor’s Efficacy for Breast Cancer. *Front Immunol.* **13**, 830158 (2022). 35444644 10.3389/fimmu.2022.830158 PMC9013822

[53] Lv, W. et al. Landscape of prognosis and immunotherapy responsiveness under tumor glycosylation-related lncRNA patterns in breast cancer. *Front Immunol.* **13**, 989928 (2022). 36189319 10.3389/fimmu.2022.989928 PMC9520571

[54] Bangarh, R. et al. Aberrant protein glycosylation: Implications on diagnosis and Immunotherapy. *Biotechnol. Adv.* **66**, 108149 (2023). 37030554 10.1016/j.biotechadv.2023.108149

[55] Lopes, N. et al. Cracking the breast cancer glyco-code through glycan-lectin interactions: targeting immunosuppressive macrophages. *Int. J. Mol. Sci*. **22** (2021). 10.3390/ijms22041972 PMC7922062 33671245

[56] Chakraborty, M. et al. Clinical relevance of glycosylation in triple negative breast cancer: a review. *Glycoconj. J.* **41**, 79–91 (2024). 38634956 10.1007/s10719-024-10151-0

[57] Bianchini, G. & Gianni, L. The immune system and response to HER2-targeted treatment in breast cancer. *Lancet Oncol.* **15**, e58–e68 (2014). 24480556 10.1016/S1470-2045(13)70477-7

[58] Herrmann, F. et al. HER-2/neu-mediated regulation of components of the MHC class I antigen-processing pathway. *Cancer Res.* **64**, 215–220 (2004). 14729627 10.1158/0008-5472.can-2522-2

[59] Mimura, K. et al. T cell recognition of HLA-A2 restricted tumor antigens is impaired by the oncogene HER2. *Int. J. Cancer* **128**, 390–401 (2011). 20715101 10.1002/ijc.25613

[60] Emens, L. A. et al. Trastuzumab emtansine plus atezolizumab versus trastuzumab emtansine plus placebo in previously treated, HER2-positive advanced breast cancer (KATE2): a phase 2, multicentre, randomised, double-blind trial. *Lancet Oncol.* **21**, 1283–1295 (2020). 33002436 10.1016/S1470-2045(20)30465-4

[61] Peraldi, P. et al. The primary cilium of adipose progenitors is necessary for their differentiation into cancer-associated fibroblasts that promote migration of breast cancer cells in vitro. *Cells* **9** (2020). 10.3390/cells9102251 PMC7601294 33049976

[62] Collinson, R. & Tanos, B. Primary cilia and cancer: a tale of many faces. *Oncogene*, 2025. 10.1038/s41388-025-03416-x PMC12095056 40301543

[63] Tong, J. et al. CDK4/6 Inhibition Suppresses p73 Phosphorylation and Activates DR5 to Potentiate Chemotherapy and Immune Checkpoint Blockade. *Cancer Res.* **82**, 1340–1352 (2022). 35149588 10.1158/0008-5472.CAN-21-3062 PMC8983601

[64] Roepman, P. et al. Microarray-based determination of estrogen receptor, progesterone receptor, and HER2 receptor status in breast cancer. *Clin. Cancer Res.* **15**, 7003–7011 (2009). 19887485 10.1158/1078-0432.CCR-09-0449

[65] Viale, G. et al. High concordance of protein (by IHC), gene (by FISH; HER2 only), and microarray readout (by TargetPrint) of ER, PgR, and HER2: results from the EORTC 10041/BIG 03-04 MINDACT trial. *Ann. Oncol.* **25**, 816–823 (2014). 24667714 10.1093/annonc/mdu026 PMC3969556

[66] Ferno, M. et al. Intra- and inter-laboratory reproducibility of estrogen and progesterone receptor enzyme immunoassay in breast cancer cytosol samples–a Swedish multicenter study. Swedish Society of Cancer Study Group. *Acta Oncol.* **36**, 793–798 (1997). 9482684 10.3109/02841869709001359

[67] Allison, K. H. et al. Estrogen and progesterone receptor testing in breast cancer: ASCO/CAP guideline update. *J. Clin. Oncol.* **38**, 1346–1366 (2020). 31928404 10.1200/JCO.19.02309

[68] Chebil, G. et al. Comparison of immunohistochemical and biochemical assay of steroid receptors in primary breast cancer–clinical associations and reasons for discrepancies. *Acta Oncol.* **42**, 719–725 (2003). 14690157 10.1080/02841860310004724

[69] Goldhirsch, A. et al. Personalizing the treatment of women with early breast cancer: highlights of the St Gallen International Expert Consensus on the Primary Therapy of Early Breast Cancer 2013. *Ann. Oncol.* **24**, 2206–2223 (2013). 23917950 10.1093/annonc/mdt303 PMC3755334

[70] Maisonneuve, P. et al. Proposed new clinicopathological surrogate definitions of luminal A and luminal B (HER2-negative) intrinsic breast cancer subtypes. *Breast Cancer Res.* **16**, R65 (2014). 24951027 10.1186/bcr3679 PMC4095689

[71] Ehinger, A. et al. Histological grade provides significant prognostic information in addition to breast cancer subtypes defined according to St Gallen 2013. *Acta Oncol.* **56**, 68–74 (2017). 27762648 10.1080/0284186X.2016.1237778

[72] Zon M, G. D., Haibe-Kains B. *MetaGxBreast: Transcriptomic Breast Cancer Datasets*. 2021.

[73] Servant, N. et al. Search for a gene expression signature of breast cancer local recurrence in young women. *Clin. Cancer Res.* **18**, 1704–1715 (2012). 22271875 10.1158/1078-0432.CCR-11-1954

[74] Yu, G. et al. clusterProfiler: an R package for comparing biological themes among gene clusters. *Omics* **16**, 284–287 (2012). 22455463 10.1089/omi.2011.0118 PMC3339379

[75] Yu, G. & He, Q.-Y. ReactomePA: an R/Bioconductor package for reactome pathway analysis and visualization. *Mol. Biosyst.* **12**, 477–479 (2016). 26661513 10.1039/c5mb00663e

[76] Szklarczyk, D. et al. The STRING database in 2023: protein-protein association networks and functional enrichment analyses for any sequenced genome of interest. *Nucleic Acids Res.* **51**, D638–d646 (2023). 36370105 10.1093/nar/gkac1000 PMC9825434

[77] Kuleshov, M. V. et al. Enrichr: a comprehensive gene set enrichment analysis web server 2016 update. *Nucleic Acids Res.* **44**, W90–W97 (2016). 27141961 10.1093/nar/gkw377 PMC4987924

[78] Teschendorff, A. E. et al. A comparison of reference-based algorithms for correcting cell-type heterogeneity in Epigenome-Wide Association Studies. *BMC Bioinforma.* **18**, 105 (2017). 10.1186/s12859-017-1511-5 PMC5307731 28193155

[79] Li, Z. & Wu, H. TOAST: improving reference-free cell composition estimation by cross-cell type differential analysis. *Genome Biol.* **20**, 190 (2019). 31484546 10.1186/s13059-019-1778-0 PMC6727351

[80] Zhang, Z. Multiple imputation with multivariate imputation by chained equation (MICE) package. *Ann. Transl. Med.* **4**, 30 (2016). 26889483 10.3978/j.issn.2305-5839.2015.12.63 PMC4731595

[81] Fine, J. P. & Gray, R. J. A proportional hazards model for the subdistribution of a competing risk. *J. Am. Stat. Assoc.* **94**, 496–509 (1999).

