# Supplementary material for: Predicting immune responsiveness in ER-positive breast cancer for personalized therapy: a population-based study
Source: NPJ Precis Oncol. 2025 Jul 23;9:250. doi: 10.1038/s41698-025-01035-z (PMC12287262; doi:10.1038/s41698-025-01035-z)
Supplement: Supplementary file 1 — Supplement 6-12 [file 41698_2025_1035_MOESM1_ESM.docx]

**Supplement**

**Figure S1. Cumulative incidence of ipsilateral breast tumor recurrence in patients treated with radiotherapy, systemic therapy, radical surgery of age <50 years or 50-65 years with histological grade III**

*These subgroups were selected based on current clinical guidelines, which recommend a radiotherapy boost in these settings due to their elevated IBTR risk. Patients were stratified by the median Immunescore within each group to assess whether immune infiltration could refine risk stratification and identify patients with a favorable prognosis despite high-risk clinical features. Cumulative incidence curves were calculated using the Fine and Gray method, and differences between groups were evaluated using Gray’s test.*

**Figure S2. Violin plot of epithelial cell states stratified by estrogen receptor status in the training cohort**

*Abundance of each epithelial cell state stratified by ER status. Violin plots show the distribution of abundance scores across epithelial states, split by ER-positive (top) and ER-negative (bottom) tumors. Points represent individual samples.*

**Figure S3. Correlation plot of epithelial cell states and clinicopathological variables in the JK biobank cohort**

*Spearman correlation tests were performed between clinicopathological variables and the epithelial cell states defined by EcoTyper. The S04 epithelial cell state was associated with aggressive clinicopathological characteristics and higher Immunescore while the S05 epithelial cell state showed opposite associations and positive correlation with ERBB2.*

**Figure S4. Correlations between EcoTyper-Defined Cell States in ER-positive Tumors of the JK Biobank Cohort**

*Spearman correlation matrix with hierarchical clustering visualizing associations between EcoTyper-defined epithelial, immune, and stromal cell states in ER-positive tumors from the JK Biobank cohort. Colors indicate the strength and direction of the correlations (blue = negative, red = positive), with white representing no correlation. Cell states were derived using EcoTyper based on bulk RNA-sequencing data. The S04 epithelial cell state (“Epi_Proinfl (S04)”), which was found to predict immune responsiveness, was positively correlated with proinflammatory immune cells spanning the innate and adaptive immune response, supporting its association with broad changes in the tumor microenvironment. The S05 epithelial cell state (“Epi_Unknown (S05)”), which predicted absence of prognostic benefit from an immune infiltrate, correlated with S05 Fibroblasts (“Fibro_Unknown1 (S05)”).*

*Abbreviations: Mono= Monocytes, Macro=Macrophages, Epi= Epithelial cells, CD4= CD4+ T-cells, Epi= Epithelial cells, Fibro= Fibroblasts, B= B-cells, Endo= Endothelial cells, Mast= Mast cells, NK= NK-cells, DC= Dendritic cells, Neut= Neutrophils, CD8= CD8+ T-cells*

**Figure S5. Boxplots showing association between the S04 and S05 cell states and the previously identified tumor ecotypes**

*The S04 epithelial cell state was associated with the CE9 and CE10 ecotypes. The S05 epithelial cell state showed a more heterogeneous distribution.*

**Figure S6. Gene set enrichment analysis for the S05 fibroblast cell state**

*The S05 fibroblast cell state was the cell state most strongly correlated with the epithelial S05 cell state which predicted immune evasive/immune-cold tumors. Gene set enrichment of the S05 fibroblast cell state revealed enrichment for cilium-related processes and endocrine therapy resistance.*

**Figure S7. Volcano plots for differentially methylated genes in the S04 (left) and S05 (right) epithelial cell states, respectively**

Volcano plot of differentially methylated genes in the epithelial cell states S04 and S05. The methylation analysis was stratified by genes predicted to be differentially methylated in the epithelial vs non-epithelial compartment. Genes predicted as differentially methylated in the epithelial compartment were analyzed further. The top 50 gene sites per cell type were included in the plot. Some sites did not correspond to known gene names and are not annotated. Methylation analysis revealed hypomethylation enrichment for immune-related processes and hypermethylation enrichment for genes in the PI3K pathway for the S04 cell state.

**Figure S8. RNA-seq quality control plot for the JK biobank cohort**

*Data quality was assessed by quality control metrics including library complexity, entropy, gene detection rate, and principal component analysis. FFPE-derived samples (batch 3) showed reduced library size and detected genes but did not form distinct clusters in the PCA space. Therefore, all samples were retained to maximize statistical power.*

**Supplementary Data S1. Demographics of the JK biobank cohort with known ER, RT, and IBTR status before imputations**

Table of the 416 tumors with known estrogen receptor (ER), radiotherapy (RT), and ipsilateral breast tumor recurrence (IBTR) status. ER status was imputed for 10 additional patients and RT imputed for 2 additional patients resulting in a total of 428 included patients post-imputation included for analysis. Refer to Methods and Supplementary Data S3 for imputations.

**Supplementary Data S2. Imputed values for the JK biobank cohort**

Missing values for continuous variables were imputed with the mice package using the Multivariate Imputation by Chained Equations (MICE) framework. A total of conducted over 10 iterations were run to ensure convergence, and a single imputed dataset was used for downstream analysis. Predictive mean matching (PMM) was applied for continuous variables, while logistic regression was used for binary or categorical variables were imputed using logistic regression models. To improve imputation accuracy, the model included clinical covariates used in survival modeling as well as s-phase fraction, year of diagnosis, and key biological scores such as Immunescore and ProliferativeIndex. PgR= Progesterone Receptor.

**Supplementary Data S3. Concordance between pathological (columns) subtypes and PAM50-defined (rows) subtypes**

HER2 status was determined using PAM50 for pathological subtypes. Non-luminal subtype was defined as ER-negative and HER2-negative with any histological grade and PgR status. Pathological subtypes were used for all analyses.

**Supplementary Data S4. Uni- and multivariable analysis of the risk of ipsilateral breast tumor recurrence in the whole JK biobank cohort**

Analysis including both ER-positive and ER-negative tumors from the JK biobank cohort. The interaction between Immunescore and ProliferativeIndex was significant in both univariable and multivariable analysis. PgR= Progesterone receptor.

**Supplementary Data S5. Flexible parametrics model for unirradiated patients with ER-positive tumors from the JK biobank cohort**

Flexible parametrics model for unirradiated patients with ER-positive tumors used to create predicted cumulative incidence curves for unirradiated patients in Figure 2. Backwards stepwise selection was performed starting with the covariates subtype, systemic treatment, radical surgery, tumor size, number of positive lymph nodes, PgR, and age. In addition, the curves for irradiated and unirradiated patients were matched by propensity scores to adjust for confounders associated with radiotherapy.

**Supplementary Data S6. Flexible parametrics model for irradiated patients with ER-positive tumors from the JK biobank cohort**

Flexible parametrics model for irradiated patients with ER-positive tumors used to create predicted cumulative incidence curves for unirradiated patients in Figure 2. Backwards stepwise selection was performed starting with the covariates subtype, systemic treatment, radical surgery, tumor size, number of positive lymph nodes, PgR, and age. In addition, the curves for irradiated and unirradiated patients were matched by propensity scores to adjust for confounders associated with radiotherapy.

**Supplementary Data S7. Gene set enrichment analysis of the S04 epithelial cell state**

Top enrichment results for the epithelial cell state S04 gene list as defined by the original EcoTyper paper. The table includes the top 10 GO, Reactome, protein nodes, and transcription factor motifs, respectively. P values were adjusted using the FDR method. Pathway enrichment analyses were performed for the S04 and S05 cell states using their respective gene sets using clusterProfiler (Gene Ontology), ReactomePA (Reactome pathways), STRINGdb (protein nodes), and enrichR] (transcription factor motifs). *Degree is used instead of adjusted p value for protein nodes and represents the number of known or predicted interactions the protein has within the STRING protein–protein interaction network.

**Supplementary Data S8. Gene set enrichment analysis of the S05 epithelial cell state**

Top enrichment results for the epithelial cell state S05 gene list as defined by the original EcoTyper paper. The table includes the top 10 GO, Reactome, protein nodes, and transcription factor motifs, respectively. P values were adjusted using the FDR method. Pathway enrichment analyses were performed for the S04 and S05 cell states using their respective gene sets using clusterProfiler (Gene Ontology), ReactomePA (Reactome pathways), STRINGdb (protein nodes), and enrichR] (transcription factor motifs). *Degree is used instead of adjusted p value for protein nodes and represents the number of known or predicted interactions the protein has within the STRING protein–protein interaction network.

**Supplementary Data S9. Differentially methylated genes for the S04 epithelial cell state in ER-positive tumors of the JK biobank cohort**

Top differentially methylated gene sites within the epithelial compartment for the epithelial cell state S04 in ER-positive tumors of the JK biobank cohort. Differentially methylated CpG sites were identified using limma and the continuous epithelial cell state S04 abundance score, adjusting for epithelial and stromal/immune proportions using EpiDISH[32]. The results were intersected with methylation sites significantly altered within epithelial compartments (FDR < 0.05) as determined by TOAST[33] to improve epithelial specificity. Enrichment analyses of the resulting gene sets were later conducted.

**Supplementary Data S10. Enrichment analysis of differentially hypomethylated genes in ER-positive tumors for the S04 (left) and S05 (right) epithelial cell states**

Differentially hypomethylated CpG sites were identified using limma, adjusting for epithelial and stromal/immune proportions using EpiDISH. The results were intersected with methylation sites significantly altered within epithelial compartments (FDR < 0.05) as determined by TOAST to improve epithelial specificity. Enrichment analyses of the resulting gene sets were conducted using clusterProfiler (Gene Ontology), ReactomePA (Reactome pathways), STRINGdb (protein nodes), and enrichR (transcription factor motifs). *Degree is used instead of adjusted p value for protein nodes and represents the number of known or predicted interactions the protein has within the STRING protein–protein interaction network.

**Supplementary Data S11. Enrichment analysis of differentially hypermethylated genes in ER-positive tumors for the S04 (left) and S05 (right) epithelial cell states**

Differentially hypermethylated CpG sites were identified using limma, adjusting for epithelial and stromal/immune proportions using EpiDISH. The results were intersected with methylation sites significantly altered within epithelial compartments (FDR < 0.05) as determined by TOAST to improve epithelial specificity. Enrichment analyses of the resulting gene sets were conducted using clusterProfiler (Gene Ontology), ReactomePA (Reactome pathways), STRINGdb (protein nodes), and enrichR (transcription factor motifs). *Degree is used instead of adjusted p value for protein nodes and represents the number of known or predicted interactions the protein has within the STRING protein–protein interaction network.

**Supplementary Data S12. Differentially methylated genes for the S05 epithelial cell state in ER-positive tumors of the JK biobank cohort**

Top differentially methylated gene sites within the epithelial compartment for the epithelial cell state S05 in ER-positive tumors of the JK biobank cohort. Differentially methylated CpG sites were identified using limma and the continuous epithelial cell state S04 abundance score, adjusting for epithelial and stromal/immune proportions using EpiDISH[32]. The results were intersected with methylation sites significantly altered within epithelial compartments (FDR < 0.05) as determined by TOAST[33] to improve epithelial specificity. Enrichment analyses of the resulting gene sets were later conducted.

**Supplementary Data S13. Demographics of the combined training cohort used to investigate epithelial cell states with immunomodulatory effects in ER-positive tumors**

Demographics of training cohort used to investigate epithelial cell states with affecting immune responsiveness in ER-positive tumors. Only ER-positive tumors were used for analysis. The endpoints used was based on availability in the following order: any recurrence, distant metastasis, local recurrence and overall survival within 10 years of follow-up. Cohorts included were CAL[1], MSK[2], NCI[3], NKI[4], UNT[5], PNC[6], STK[7], STNO2[8], TCGA[9], TRANSBIG[10], DFHCC[11], UPP[12], VDX[13], Servant[14], DUKE[15], EMC2[16], GSE58644[17], GSE25066[18], UNC[19], MAINZ[20], METABRIC[21].

**Supplementary Data S14. Propensity score model used to balance irradiated vs unirradiated patients for flexible parametric models**

Propensity score model for the likelihood of receiving radiotherapy. The model was fitted using a logistic regression model with radiotherapy as the dependent variable. Backward stepwise selection was applied to retain only those covariates significantly associated with radiotherapy receipt, thereby reducing model complexity while preserving predictive performance.

**Figure S1. Cumulative incidence of ipsilateral breast tumor recurrence (IBTR) in patients treated with radiotherapy, systemic therapy, and radical breast-conserving surgery, stratified by Immunescore. Two high-risk groups were analyzed: (1) patients aged <50 years, and (2) patients aged 50–65 years with histological grade III tumors**


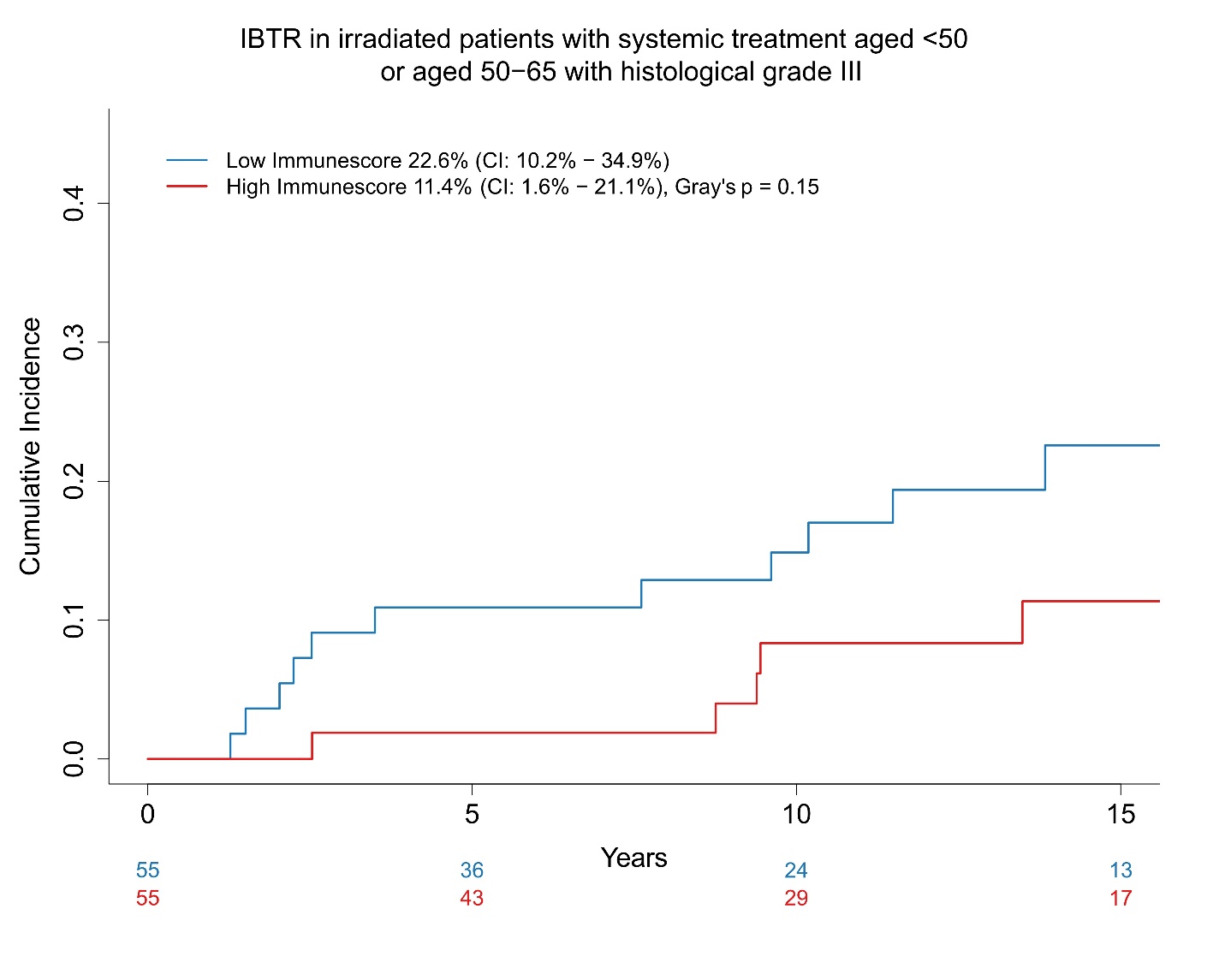


*These subgroups were selected based on current clinical guidelines, which recommend a radiotherapy boost in these settings due to their elevated IBTR risk. Patients were stratified by the median Immunescore within each group to assess whether immune infiltration could refine risk stratification and identify patients with a favorable prognosis despite high-risk clinical features. Cumulative incidence curves were calculated using the Fine and Gray method, and differences between groups were evaluated using Gray’s test.*

**Figure S2. Violin plot of epithelial cell states stratified by estrogen receptor status in the training cohort**


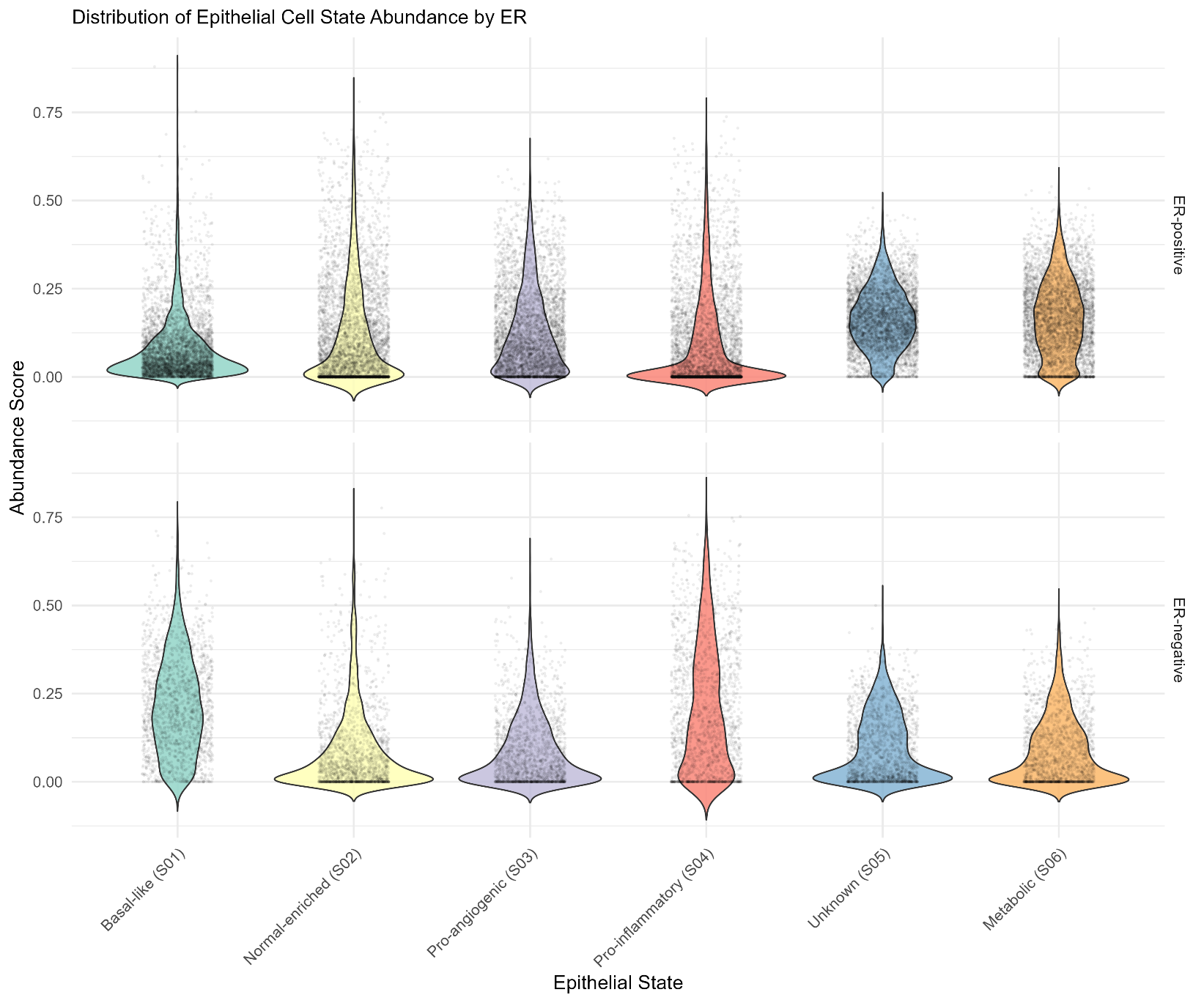


*Abundance of each epithelial cell state stratified by ER status. Violin plots show the distribution of abundance scores across epithelial states, split by ER-positive (top) and ER-negative (bottom) tumors. Points represent individual samples.*

**Figure S3. Correlation plot of epithelial cell states and clinicopathological variables in the JK biobank cohort**


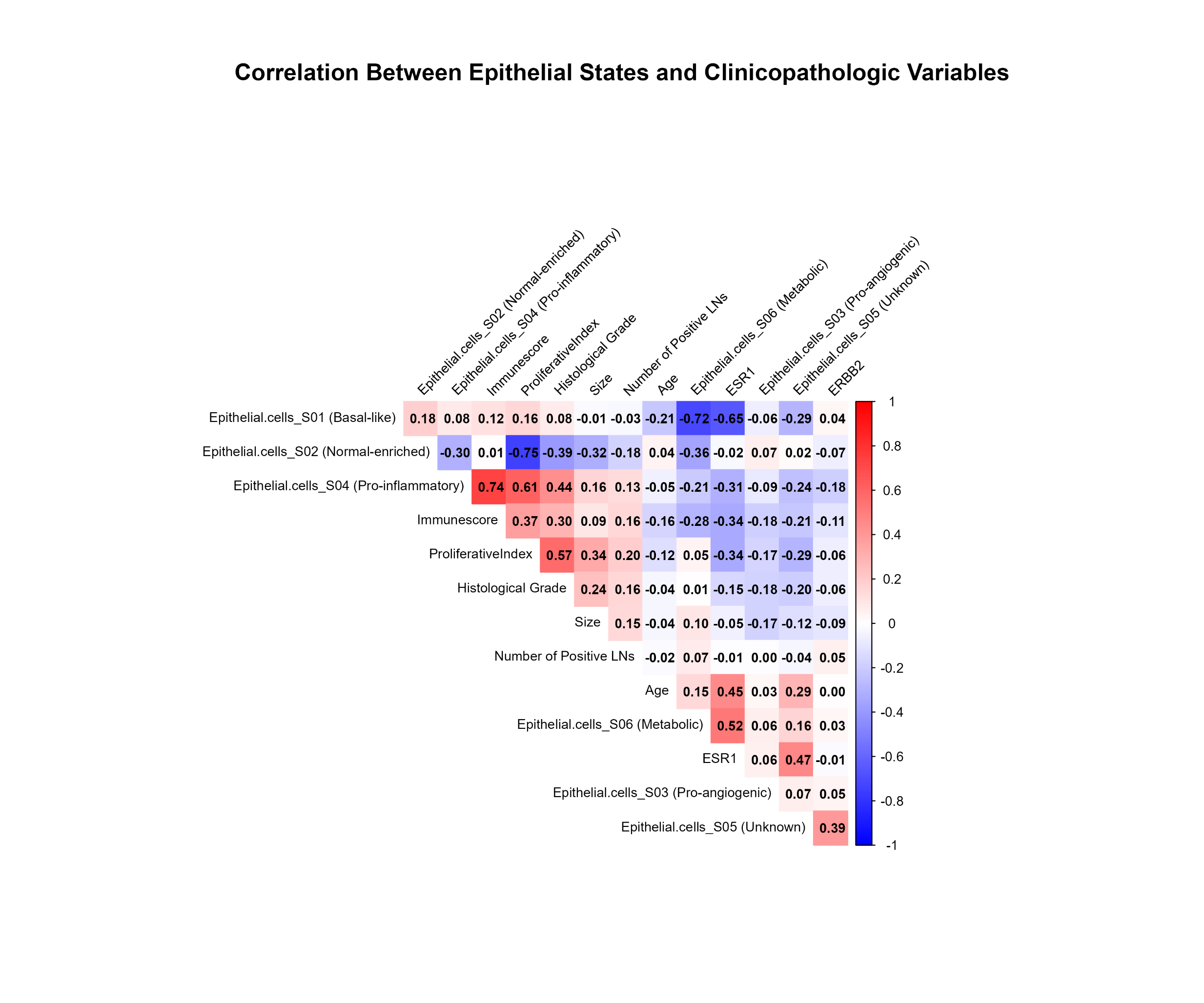


*Spearman correlation tests were performed between clinicopathological variables and the epithelial cell states defined by EcoTyper. The S04 epithelial cell state, predictive of immune responsiveness, was associated with aggressive clinicopathological characteristics and higher Immunescore while the S05 epithelial cell state, associated with lack a benefit from an immune infiltrate, showed opposite associations and positive correlation with ERBB2.*

**Figure S4. Correlations between EcoTyper-Defined Cell States in ER-positive Tumors of the JK Biobank Cohort**


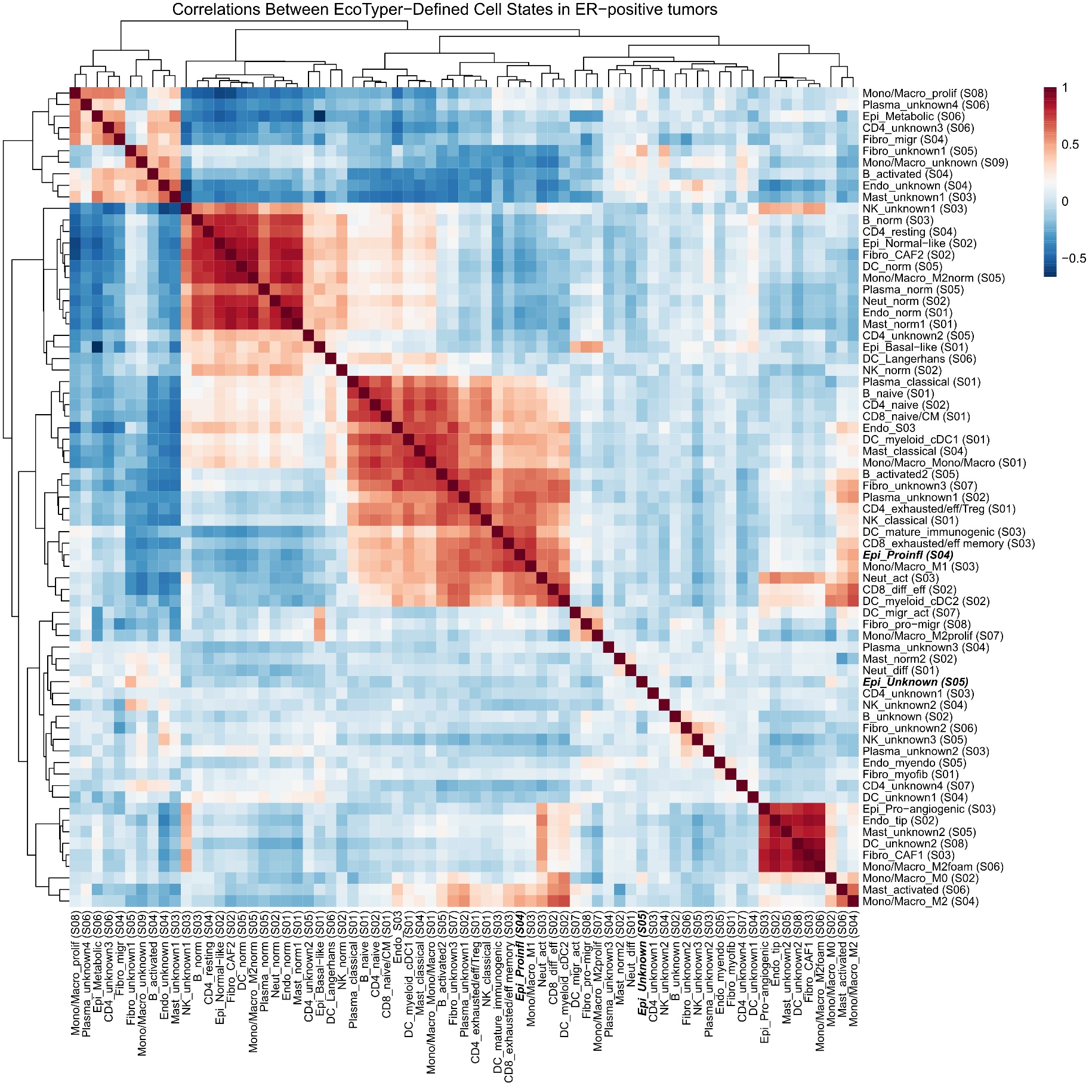


*Spearman correlation matrix with hierarchical clustering visualizing associations between EcoTyper-defined epithelial, immune, and stromal cell states in ER-positive tumors from the JK Biobank cohort. Colors indicate the strength and direction of the correlations (blue = negative, red = positive), with white representing no correlation. Cell states were derived using EcoTyper based on bulk RNA-sequencing data. The S04 epithelial cell state (“Epi_Proinfl (S04)”), which was found to predict immune responsiveness, was positively correlated with proinflammatory immune cells spanning the innate and adaptive immune response, supporting its association with broad changes in the tumor microenvironment. The S05 epithelial cell state (“Epi_Unknown (S05)”), which predicted absence of prognostic benefit from an immune infiltrate, correlated with S05 Fibroblasts (“Fibro_Unknown1 (S05)”).*

*Abbreviations: Mono= Monocytes, Macro=Macrophages, Epi= Epithelial cells, CD4= CD4+ T-cells, Epi= Epithelial cells, Fibro= Fibroblasts, B= B-cells, Endo= Endothelial cells, Mast= Mast cells, NK= NK-cells, DC= Dendritic cells, Neut= Neutrophils, CD8= CD8+ T-cells***Figure S5. Boxplots showing association between the S04 and S05 cell states and the previously identified tumor ecotypes**


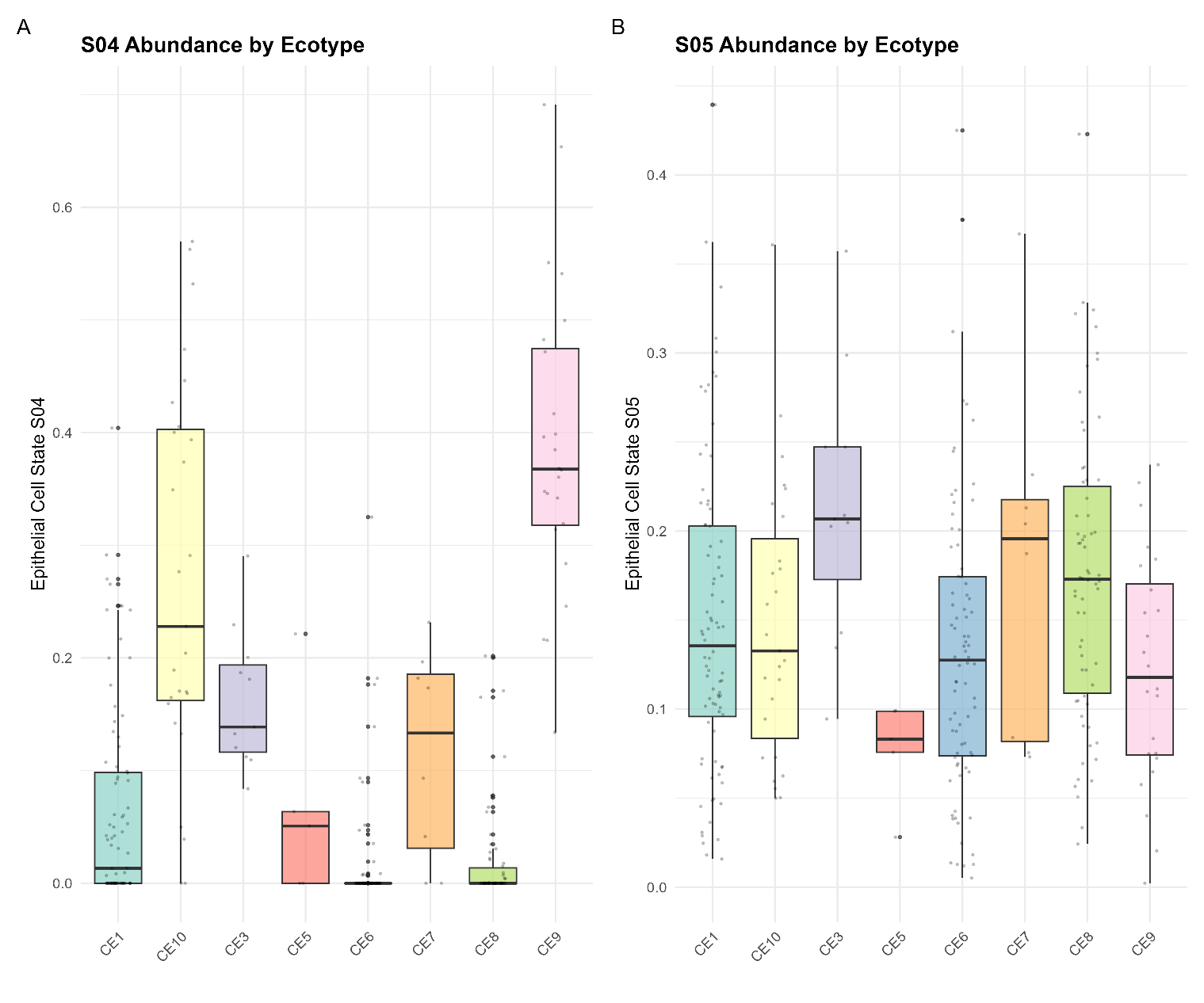


*The S04 epithelial cell state was associated with the CE9 and CE10 ecotypes. The S05 epithelial cell state showed a more heterogeneous distribution.*

**Figure S6. Gene set enrichment analysis for the S05 fibroblast cell state**


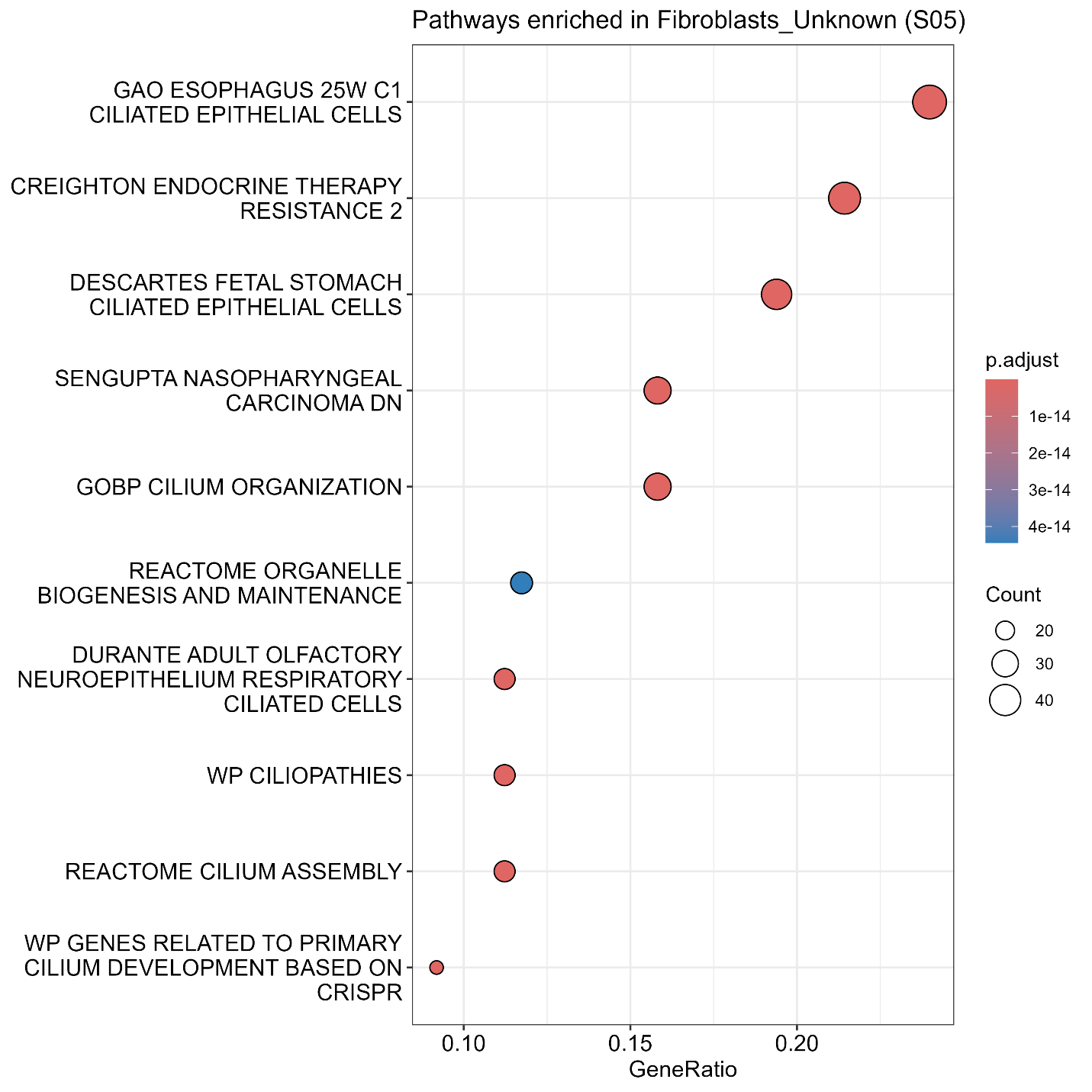


*The S05 fibroblast cell state was the cell state most strongly correlated with the epithelial S05 cell state which predicted immune evasive/immune-cold tumors. Gene set enrichment of the S05 fibroblast cell state revealed enrichment for cilium-related processes and endocrine therapy resistance.*


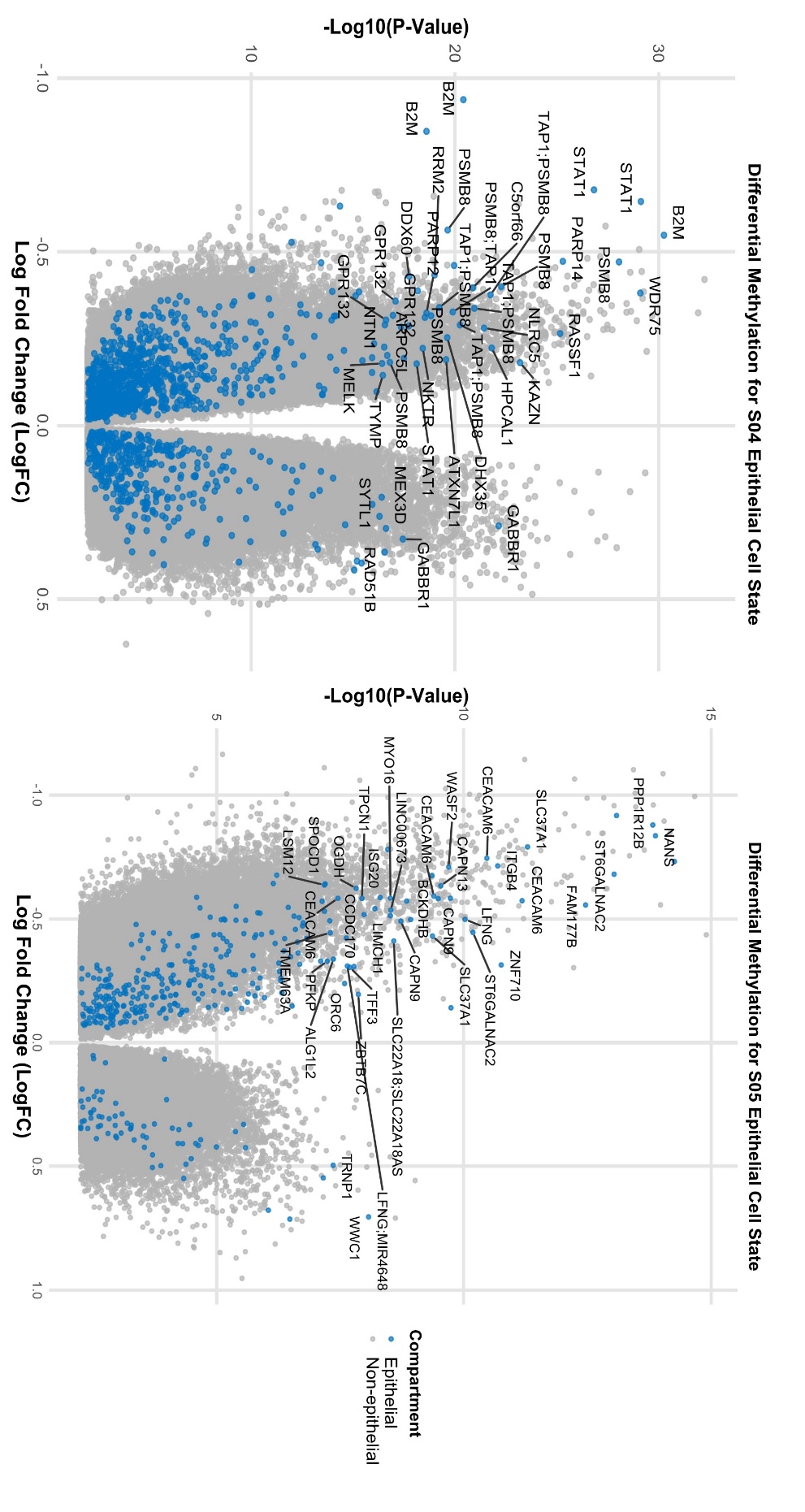
**Figure S7. Volcano plots for differentially methylated genes in the S04 (left) and S05 (right) epithelial cell states, respectively**

Volcano plot of differentially methylated genes in the epithelial cell states S04 and S05. The methylation analysis was stratified by genes predicted to be differentially methylated in the epithelial vs non-epithelial compartment. Genes predicted as differentially methylated in the epithelial compartment were analyzed further. The top 50 gene sites per cell type were included in the plot. Some sites did not correspond to known gene names and are not annotated. Methylation analysis revealed hypomethylation enrichment for immune-related processes and hypermethylation enrichment for genes in the PI3K pathway for the S04 cell state.

**Figure S8. Quality control plot for the JK biobank RNA sequencing data**


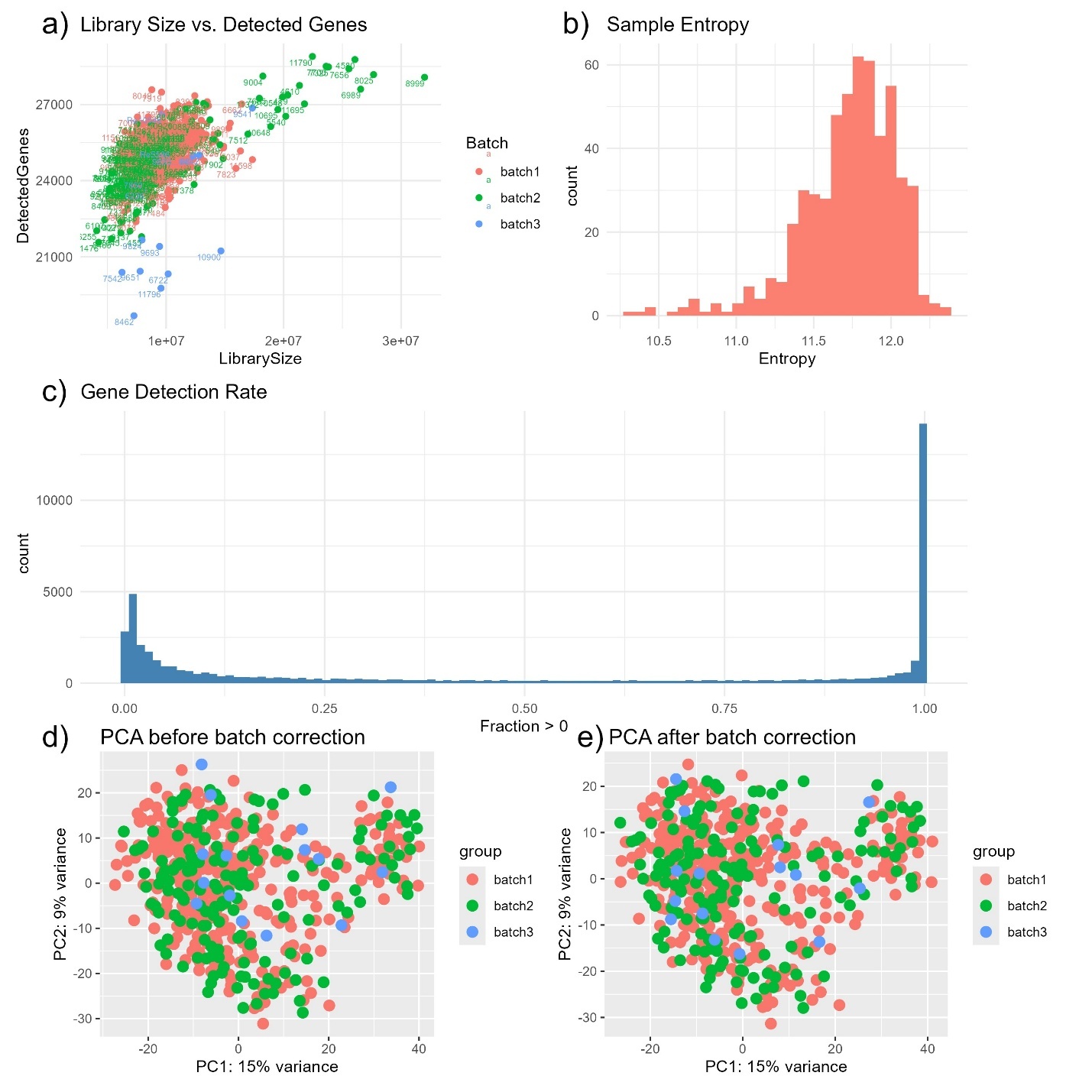


*Data quality was assessed by quality control metrics including library complexity, entropy, gene detection rate, and principal component analysis. FFPE-derived samples (batch 3) showed reduced library size and detected genes but did not form distinct clusters in the PCA space. Therefore, all samples were retained to maximize statistical power.*

1. Chin, K., et al., *Genomic and transcriptional aberrations linked to breast cancer pathophysiologies.* Cancer Cell, 2006. **10**(6): p. 529-41.

2. Minn, A.J., et al., *Genes that mediate breast cancer metastasis to lung.* Nature, 2005. **436**(7050): p. 518-24.

3. Sotiriou, C., et al., *Breast cancer classification and prognosis based on gene expression profiles from a population-based study.* Proc Natl Acad Sci U S A, 2003. **100**(18): p. 10393-8.

4. van de Vijver, M.J., et al., *A gene-expression signature as a predictor of survival in breast cancer.* N Engl J Med, 2002. **347**(25): p. 1999-2009.

5. Sotiriou, C., et al., *Gene expression profiling in breast cancer: understanding the molecular basis of histologic grade to improve prognosis.* J Natl Cancer Inst, 2006. **98**(4): p. 262-72.

6. Dedeurwaerder, S., et al., *DNA methylation profiling reveals a predominant immune component in breast cancers.* EMBO Mol Med, 2011. **3**(12): p. 726-41.

7. Pawitan, Y., et al., *Gene expression profiling spares early breast cancer patients from adjuvant therapy: derived and validated in two population-based cohorts.* Breast Cancer Res, 2005. **7**(6): p. R953-64.

8. Sorlie, T., et al., *Repeated observation of breast tumor subtypes in independent gene expression data sets.* Proc Natl Acad Sci U S A, 2003. **100**(14): p. 8418-23.

9. *Comprehensive molecular portraits of human breast tumours.* Nature, 2012. **490**(7418): p. 61-70.

10. Desmedt, C., et al., *Strong time dependence of the 76-gene prognostic signature for node-negative breast cancer patients in the TRANSBIG multicenter independent validation series.* Clin Cancer Res, 2007. **13**(11): p. 3207-14.

11. Li, Y., et al., *Amplification of LAPTM4B and YWHAZ contributes to chemotherapy resistance and recurrence of breast cancer.* Nat Med, 2010. **16**(2): p. 214-8.

12. Miller, L.D., et al., *An expression signature for p53 status in human breast cancer predicts mutation status, transcriptional effects, and patient survival.* Proc Natl Acad Sci U S A, 2005. **102**(38): p. 13550-5.

13. Wang, Y., et al., *Gene-expression profiles to predict distant metastasis of lymph-node-negative primary breast cancer.* Lancet, 2005. **365**(9460): p. 671-9.

14. Servant, N., et al., *Search for a gene expression signature of breast cancer local recurrence in young women.* Clin Cancer Res, 2012. **18**(6): p. 1704-15.

15. Bild, A.H., et al., *Oncogenic pathway signatures in human cancers as a guide to targeted therapies.* Nature, 2006. **439**(7074): p. 353-7.

16. Bos, P.D., et al., *Genes that mediate breast cancer metastasis to the brain.* Nature, 2009. **459**(7249): p. 1005-9.

17. Tofigh, A., et al., *The prognostic ease and difficulty of invasive breast carcinoma.* Cell Rep, 2014. **9**(1): p. 129-142.

18. Hatzis, C., et al., *A genomic predictor of response and survival following taxane-anthracycline chemotherapy for invasive breast cancer.* Jama, 2011. **305**(18): p. 1873-81.

19. Prat, A., et al., *Phenotypic and molecular characterization of the claudin-low intrinsic subtype of breast cancer.* Breast Cancer Res, 2010. **12**(5): p. R68.

20. Schmidt, M., et al., *The humoral immune system has a key prognostic impact in node-negative breast cancer.* Cancer Res, 2008. **68**(13): p. 5405-13.

21. Curtis, C., et al., *The genomic and transcriptomic architecture of 2,000 breast tumours reveals novel subgroups.* Nature, 2012. **486**(7403): p. 346-52.
